# Tetrathiafulvalene – a redox-switchable building block to control motion in mechanically interlocked molecules

**DOI:** 10.3762/bjoc.14.190

**Published:** 2018-08-20

**Authors:** Hendrik V Schröder, Christoph A Schalley

**Affiliations:** 1 Institut für Chemie und Biochemie, Organische Chemie, Freie Universität Berlin, Takustraße 3, 14195 Berlin, Germany

**Keywords:** artificial molecular machines, mechanically interlocked molecules, molecular switches, supramolecular chemistry, tetrathiafulvalene

## Abstract

With the rise of artificial molecular machines, control of motion on the nanoscale has become a major contemporary research challenge. Tetrathiafulvalenes (TTFs) are one of the most versatile and widely used molecular redox switches to generate and control molecular motion. TTF can easily be implemented as functional unit into molecular and supramolecular structures and can be reversibly oxidized to a stable radical cation or dication. For over 20 years, TTFs have been key building blocks for the construction of redox-switchable mechanically interlocked molecules (MIMs) and their electrochemical operation has been thoroughly investigated. In this review, we provide an introduction into the field of TTF-based MIMs and their applications. A brief historical overview and a selection of important examples from the past until now are given. Furthermore, we will highlight our latest research on TTF-based rotaxanes.

## Introduction

Undoubtedly, the exploration of nature’s molecular machines in the last century led to a paradigm change of how we think about working and organization processes on the molecular level [1–3]. Inspired by the way how energy and concentration gradients control repetitive motions of these biological nanomachines, researchers have been seeking for synthetic analogues, i.e., artificial molecular machines (AMMs), with the ultimate goal to convert energy into directional mechanical motion on the nanoscale [4–6]. The field of AMMs beautifully coalesces the desire of reproducing the versatile functions of nature’s biomachinery and the miniaturization of macroscopic technical devices made by man. Although the field of AMMs is relatively young, the Nobel Prize in 2016 for Jean-Pierre Sauvage [7], Sir J. Fraser Stoddart [8], and Bernard L. Feringa [9] "for the design and synthesis of molecular machines" is an outstanding appreciation of the public and scientific community.

Mechanically interlocked molecules (MIMs) such as rotaxanes [10] or catenanes [11] are ideally suited for the construction of AMMs. In comparison to covalently linked molecules, the mechanical bond provides cohesive supramolecular assemblies with unique properties and a high flexibility and mobility of the subcomponents in a small molecular space. To control molecular motion, one of the most important construction principles to transform a simple MIM into an AMM is to implement a switching unit into the molecular framework, which is reversibly addressable by external stimuli [12–13]. A variety of different stimuli to control MIMs has been reported ranging, for example, from physical stimuli such as electrons, light, temperature, pressure, or magnetism to chemical stimuli such as acids/bases, ions, additives, or solvent changes [14]. However, the latter class of stimuli bears the disadvantage to produce chemical “waste” which creates the challenging task to constantly add and remove material to and from the system, if a repetitive operation is desired. Therefore, a “clean” stimulus is often preferred.

**Figure 1:**
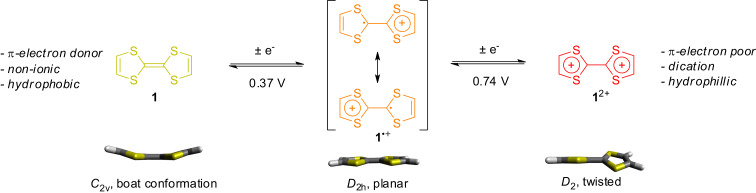
The two one-electron oxidation reactions of tetrathiafulvalene (TTF, **1**) and the corresponding property changes.

One of the most frequently used and thoroughly characterized “clean” switches to control molecular motion of MIMs is tetrathiafulvalene (TTF, **1**) and its derivatives (Figure 1). TTF is a redox-switchable organosulfur compound, which exhibits ideal properties for the electrochemical operation of MIMs. Several excellent reviews on the use of TTF in other supramolecular systems such as macrocycles, cages, and receptor molecules are already available [15–21]. In this review, motifs of construction and working principles of TTF-based MIMs in the past and current literature are summarized and milestones of their development are discussed. In the first part, we will briefly describe how TTF evolved into a key building block for switchable supramolecular architectures and which synthetic breakthroughs enabled this development. We also aim for a tutorial introduction to readers new to the field of TTF-switchable MIMs.

## Review

### 1. Tetrathiafulvalene – an (almost) perfect molecular switch

Whereas inorganic chemists are used to commonly handle metal-based compounds in different oxidation states, only a small selection of organic molecules [22] can be reversibly oxidized or reduced without chemical side reactions or decomposition. TTF is perhaps one of the most popular examples and exists as a classical Weitz type redox system [22] in three different stable oxidation states. The stability of TTF, both in solution and in the solid state [23], makes it an ideal molecular switch.

A first one-electron oxidation [23] converts neutral TTF (**1**) into the radical-cationic species **1**^●+^ (Figure 1). The TTF radical cation is one of the rare organic radicals that are long-term stable and even isolable. A second oxidation step yields the dication **1**^2+^. Both redox-transitions are fully reversible and have surprisingly low oxidation potentials (0.37 and 0.74 V vs Ag/AgCl in CH_3_CN) [24], which enable an easily achievable electrochemical switching under ambient conditions. The stability of all oxidation states – even in the presence of air and moisture – is crucial for the efficient operation and characterization of TTF-based MIMs on a suitable laboratory timescale.

The observed stability of the two oxidation states can be explained by the stepwise aromatization of the TTF system. In the neutral state, TTF consists of two pro-aromatic 1,3-dithiolylidene rings which are connected by a C=C double bond. The first oxidation converts one ring into an aromatic 6π-electron system, which is further stabilized by a mixed-valence resonance structure. The second oxidation yields two aromatic 1,3-dithiolium cations (2 × 6π electrons) which are connected by a C–C single bond.

The change of the electronic structure is also accompanied by conformational changes [25–26] of the TTF skeleton. Neutral TTF has a boat-shaped structure with *C*_2_*_v_* symmetry. In the radical-cation state, TTF^●+^ planarizes into a *D*_2_*_h_*-symmetric structure due to its partial aromatization. This property change is widely used to induce cofacial intermolecular stacking interactions. Finally, the TTF^2+^ dication adopts a twisted conformation with *D*_2_ symmetry.

In the neutral state, TTF is a strong π-donor molecule, a property which is used in a plethora of charge-transfer materials and molecules [27]. In supramolecular chemistry [28] and for the construction of MIMs [29], the good π-donor properties of TTF are frequently used to template donor–acceptor complexes with π-electron deficient macrocycles. If TTF undergoes oxidation, the π-donating effect decreases, whereas the TTF^2+^ dication can be considered as a π-electron-poor molecule.

The electrochemical switching of a TTF unit and the change of electronic or conformational properties not necessarily results in a mechanical motion of a MIM. A prerequisite is that at least one of the above-mentioned properties of TTF is interacting with other parts of the MIM. If this property is changed, the previous conformation of the MIM might become unstable and initiates a molecular motion. This simple principle of bistability has been used to create a variety of different switchable TTF-based supramolecular architectures with many versatile applications.

**Figure 2:**
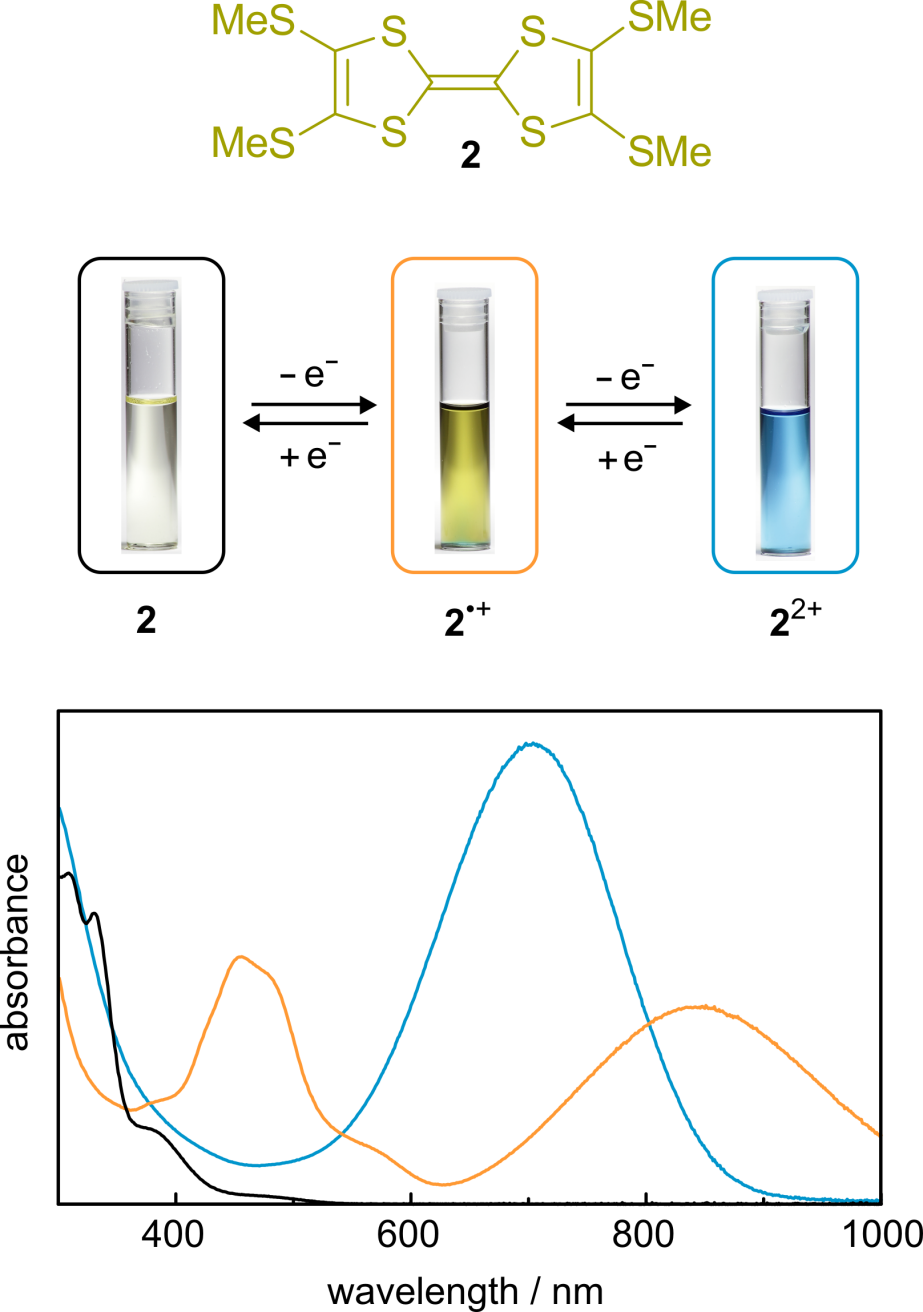
UV–vis spectra and photographs of TTF **2** in its three stable oxidation states (black line = **2**, orange line = **2**^●+^, blue line = **2**^2+^).

On the macroscopic as well as on the molecular level, even the most efficient switch is useless, if no observable output is generated which helps to detect the switching process [30]. A simple “read out” is provided by the optical properties of TTF in its different switching states. For example, UV–vis spectra of the TTF derivative **2** in the neutral, radical-cation, and dication state are shown in Figure 2. The spectrum of **2** shows only weak absorption above 350 nm which results in a pale yellow solution. The lowest-energy band is the HOMO→LUMO transition of the molecule. The radical-cation **2**^●+^ exhibits two strong absorption bands (≈450 and 800 nm), which yield an orange-brown solution. Initially, the low-energy band of **2**^●+^ between 600–1000 nm was interpreted as a signature for an unusually stable TTF dimer [31]. However, later investigations showed that this band is an intrinsic SOMO-1→SOMO transition in the **2**^●+^ radical cation [32]. The dication **2**^2+^ shows a strong band at ≈700 nm which results in a deep-blue solution. These strong color changes differ for differently substituted TTF derivatives and make it very easy to follow the electrochemical switching of TTF, even with the naked eye.

Other optical properties which are very helpful for observing the molecular switching in MIMs are charge-transfer bands. The π-donor TTF can form donor–acceptor complexes with π-electron-poor aromatic compounds often indicated by a green color of the solution [33]. Therefore, the assembly and disassembly of these complexes in solution can be easily traced by the emergence and fading of these characteristic charge-transfer bands.

**Figure 3:**
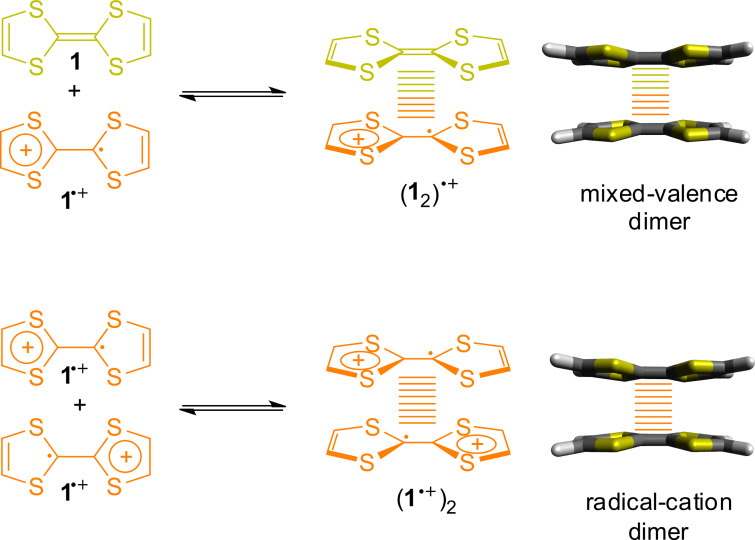
Structure and conformations of two TTF dimers in solution, the mixed-valence and the radical-cation dimer.

Another outstanding feature of TTFs is that their radical cations can reversibly form cofacial dimers (Figure 3) [34–36]. The two monomers **1** and **1**^●+^ spontaneously self-assemble into a so-called mixed-valence dimer (**1**_2_) ^●+^. A mixed-valence dimer can be identified by splitting of the first TTF oxidation potential into two distinguishable waves. This change in redox behavior can be followed by electrochemical methods such as cyclic voltammetry. Another indication for a mixed-valence dimer interaction can be an emergent low-energy absorption band, usually in the NIR region. Both monomers show usually no absorption in this region.

The radical-cation dimer (**1**^●+^)_2_ instead forms from two **1**^●+^ radical cations and exhibits a very unusual binding situation. Whereas both monomers are paramagnetic radicals, the resulting dimer has a diamagnetic character due to radical pairing. Although the distance of ≈3.5 Å between the two **1**^●+^ molecules in the dimer is considerably large in comparison to a C–C bond (≈1.5 Å), the interaction can be considered as a type of multi-centered two-electron bond with covalent character. This type of radical-cation dimer is often called a π-dimer or “pimer” and its formation “pimerization”. The radical-cation dimer can be spectroscopically identified by characteristic blue shifts of TTF^●+^ absorption bands. These “Davydov shifts” are a result of the H-aggregate-type arrangement in the dimer [34]. Furthermore, the equilibrium between a paramagnetic monomer and a diamagnetic dimer makes the use of electron paramagnetic resonance (EPR) spectroscopy ideal to follow the dimerization process [36].

Mixed-valence and radical-cation interactions in the solid state are sometimes described as “conductive” and “isolating” form, respectively. However, in solution both dimers display very low stabilities with dimerization energies of only a few kJ mol^−1^ at room temperature [36]. Therefore, these weakly associated dimers are virtually absent at ambient conditions in solution.

A strategy to stabilize the mixed-valence and radical-cation dimer even at room temperature and to overcome the entropic penalty of their formation is to facilitate a spatial proximity of two or more TTF units by a suitable covalent link [37–38]. This pre-organization can also be generated in supramolecular complexes with confined spaces which provide a very high local concentration and shift the equilibrium towards the dimer side. The use of TTF dimerization has been recognized lately as additional possibility to drive motion in MIMs. Recent examples will be discussed in the following section.

### 2. Evolution of TTF into a key building block in switchable molecular systems

After the first syntheses of native TTF in the early 1970s researches quickly noticed the outstanding electronic properties of this molecule [23,39]. One of the first observations with major impact was the unusual conducting behavior of oxidized TTF salts [40]. The discovery that TTF and the electron-deficient molecule tetracyanoquinodimethane (TCNQ) form charge-transfer salts [41] with the uncommon motif of “segregated stacks” [42] enabled numerous investigations of TTF salts regarding their application in molecular electronics [27,43], organic metals [44], or narrow-band semiconductors [45].

As often in chemical research, major synthetic or analytic breakthroughs are needed to open pathways towards new concepts and applications. Despite the intensive research on TTF during the 70s and 80s, the incorporation of TTF into molecular systems using simple organic chemistry procedures was still challenging at that time.

**Figure 4:**
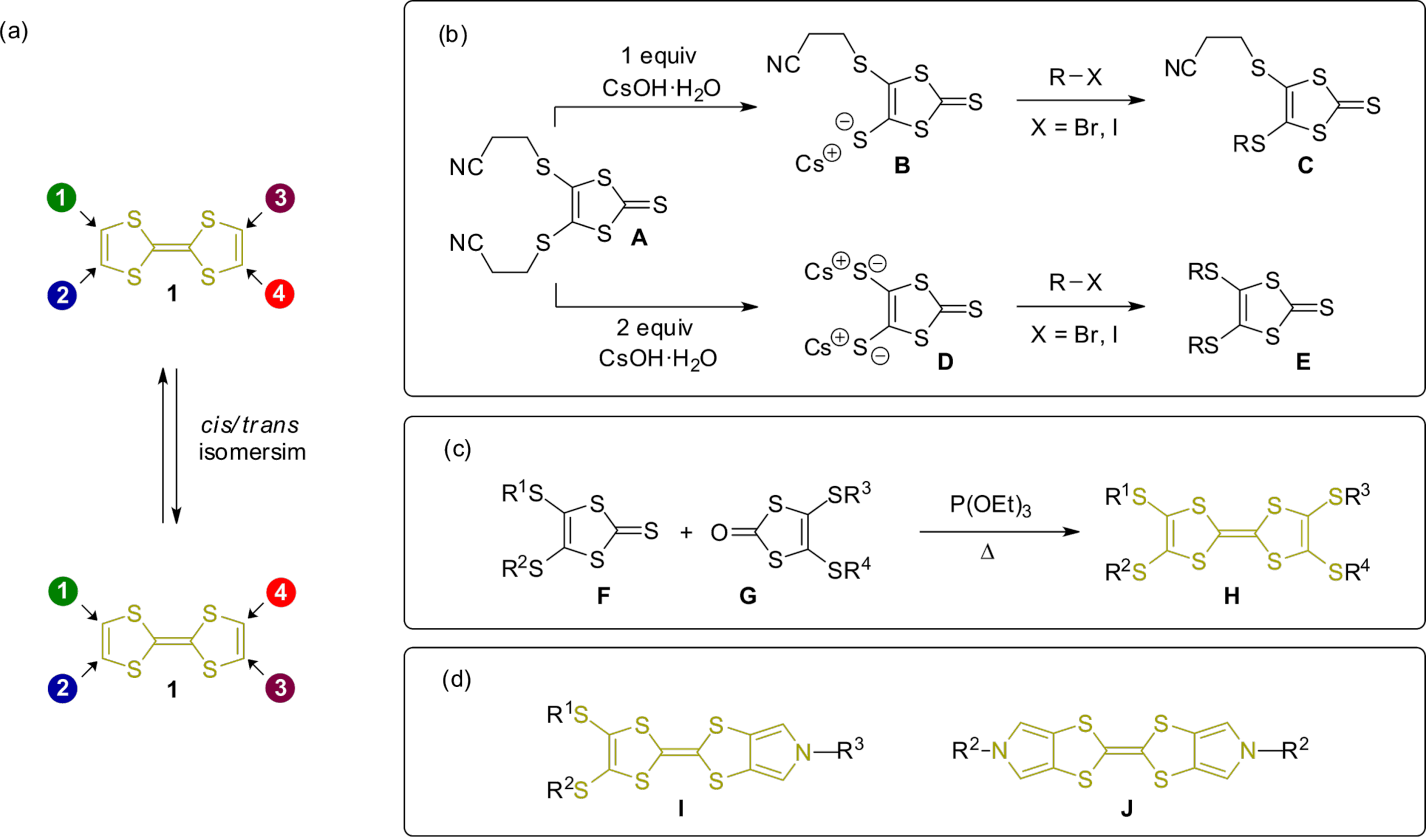
(a) The isomerism problem of TTF. (b)–(d) Major synthetic breakthroughs for the construction of TTF-based supramolecular architectures: (b) Stepwise deprotection/alkylation, (c) phosphite-mediated heterocoupling, and (d) pyrrolo-annulated TTF derivatives **I** and **J**.

One of the major synthetic breakthroughs was thus the use of cyanoethyl protective groups for TTF thiolates (Figure 4) [46–47]. Treatment of cyanoethyl-protected 1,3-dithiol-thiolates **A** with one or two equivalents of a strong base such as CsOH yields quantitatively the corresponding cesium thiolates **B** and **D** which are quite stable under standard Schlenk conditions. Addition of an alkyl halide can attach a broad range of different substituents. The cyanoethyl group allows a sequential deprotection and alkylation of 1,3-dithiole-2-thiones **C** and **E** and the corresponding TTF molecules derived from them often in very good yields. An additional strategy to obtain non-symmetrically substituted TTF derivatives is the stepwise reaction of TTF tetrathiolate with different electrophiles [48].

Another important synthetic advance is the phosphite-mediated heterocoupling of 1,3-dithiol-2-thiones **F** and 1,3-dithiol-2-ones **G** which provides efficient access to TTFs with two differently substituted 1,3-dithiol rings in an efficient way [49]. Simple heating of both monomers (ketone and thioketone) in P(OMe)_3_ or P(OEt)_3_ yields the desired hetero dimers **H** often in good yields. In combination with transchalcogenation reactions [50], which allow the transformation of 1,3-dithiol-2-thiones into the corresponding ketones in excellent yields, various types of non-symmetrically substituted TTF moieties can be implemented into organic systems.

However, a synthetic problem which was still intricate is caused by the four substitution sites of the TTF unit, which result in a mixture of *cis* and *trans* isomers, if two different substituents are attached to either one of the two 1,3-dithiolylidene rings. Isomerization can be promoted by trace amounts of acid [51–52] or photochemically [53]. The interconversion usually prevents a sufficient separation of the two isomers on the laboratory timescale. However, substitution of the TTF molecule by electron-withdrawing groups can stabilize the isomers [54] and a separation becomes possible. In particular when it comes to the complex intertwined structure of MIMs, an isolable pure compound is often necessary for a thorough characterization and investigation of their switching properties. One solution to the isomer problem is the introduction of mono- or bipyrrolo-annulated TTF derivatives **I** and **J** [55–57]. The incorporation of these symmetric species into MIMs often circumvents complex isomeric mixtures.

### 3. Pseudorotaxanes and inclusion complexes: on the way to TTF-based MIMs

Pseudorotaxanes have the general form of a molecular thread encircled by a macrocycle. The difference to rotaxanes is that the axle does not have bulky stopper groups that prevent the deslipping of the wheel. Thus, a pseudorotaxane forms by non-covalent interactions between host and guest without a mechanical bond. Pseudorotaxanes are important precursors of MIMs from which the construction of rotaxanes is achieved by stoppering reactions, while catenanes can be made by macrocyclization of the pseudorotaxane thread. Therefore, we discuss in the following section reports of important pseudorotaxanes and inclusion complexes that contributed to major developments of TTF-based MIMs and AMMs.

**Figure 5:**
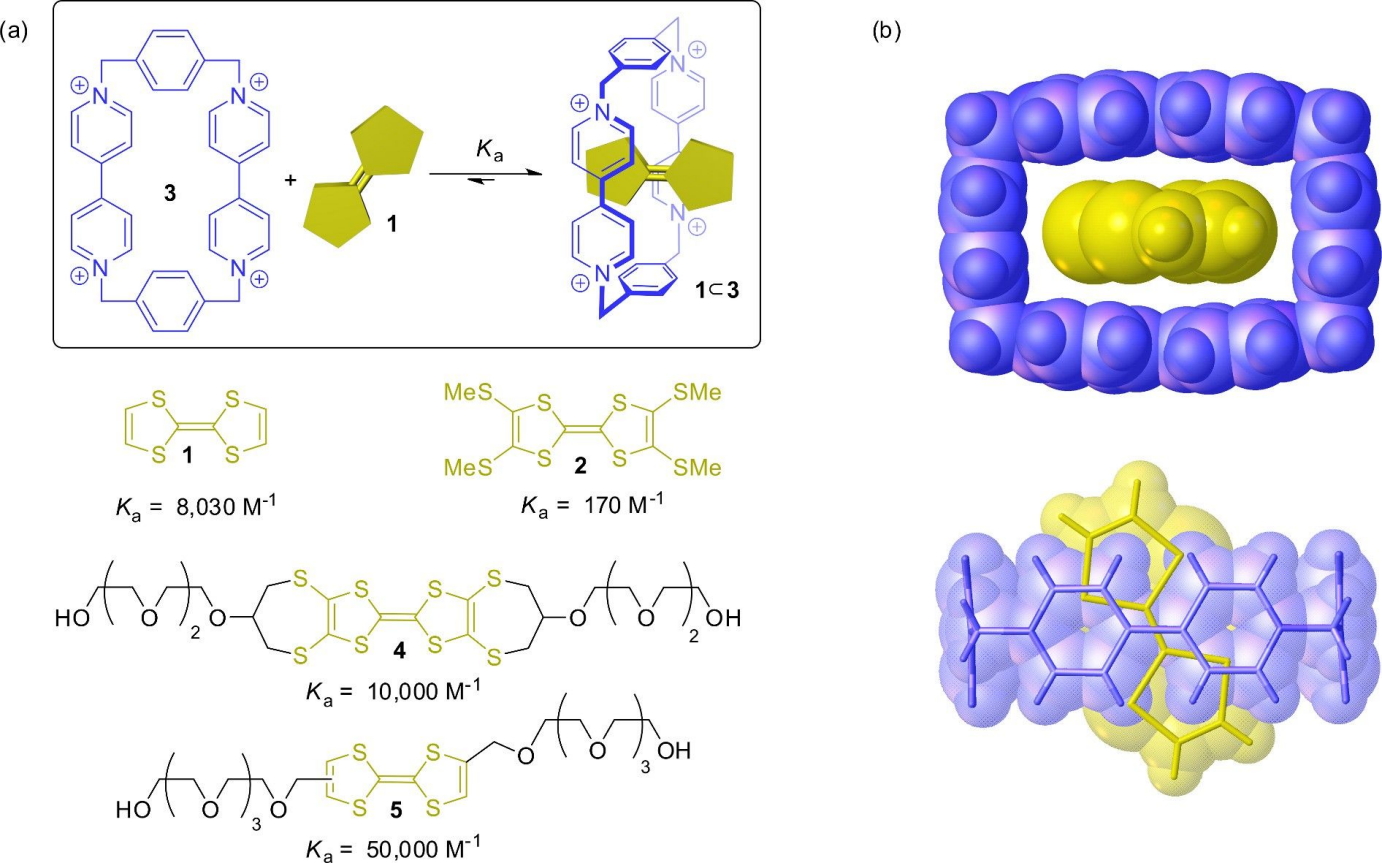
(a) Host–guest equilibrium between π-electron-poor cyclophane **3** and different TTFs with their corresponding association constants in CH_3_CN. (b) Crystal structure of host–guest complex **1**
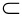
**3** [33]. Solvent molecules and counterions are omitted for clarity.

The first TTF-based pseudorotaxane was reported by Stoddart and Williams in 1991 (Figure 5) [33]. At this time, they investigated the host–guest properties of the π-electron-poor cyclophane cyclobis(paraquat-*p*-phenylene) (**3**) in form of the tetrakis(hexafluorophosphate) salt [58]. The square-shaped host complexes π-electron-rich aromatic compounds such as dihydroxynaphthalenes or dihydroxybenzenes. The π-donor TTF (**1**) also forms a 1:1 complex **1**
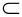
**3** with this host molecule as shown in solution experiments and by crystallography. The complex formation is immediately visible by an emergent green color of these solutions due to the donor–acceptor interaction.

In later reports, differently substituted TTF derivatives as for example **2**, **4**, and **5** have been investigated towards their binding to host **3** [24,59–63]. π-Electron-rich TTFs form significantly stronger donor–acceptor complexes as π-electron-poor TTFs. However, also the type of substituent on the TTF moiety plays a role in terms of weak secondary binding interactions such as hydrogen bonds [63]. For example TTF **5** which is substituted by ethylene glycol chains displays a high association constant of *K*_a_ = 50,000 M^−1^ in acetonitrile. Additionally, extended π-surfaces [64] of TTF derivatives can have a stabilizing effect upon complexation.

**Figure 6:**
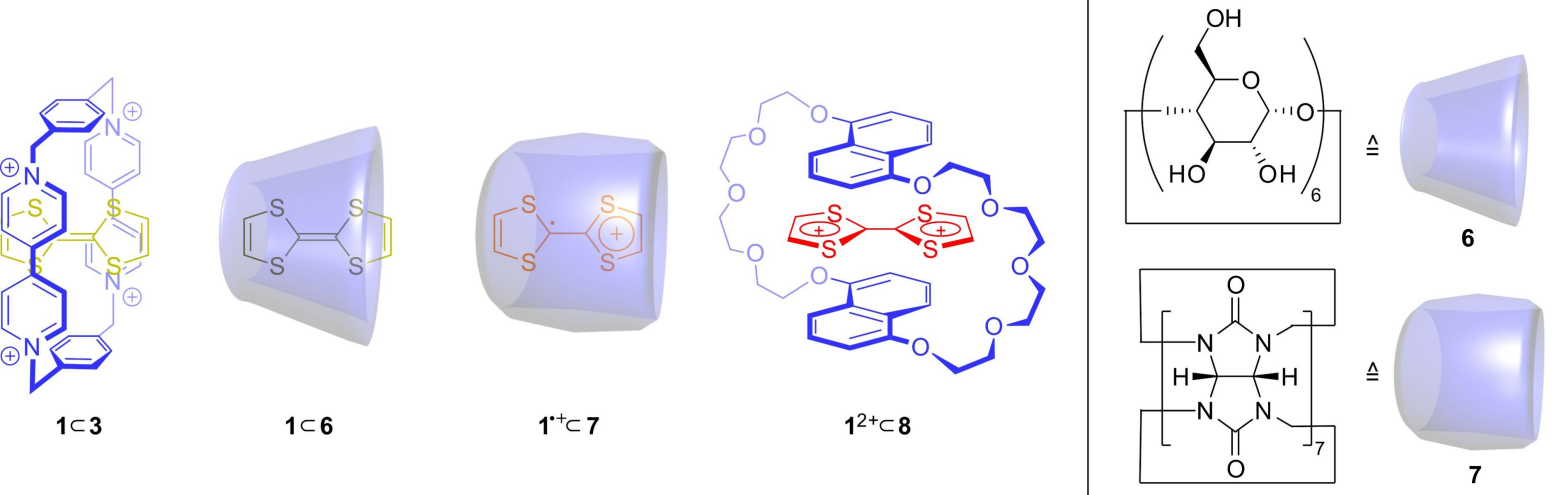
TTF complexes with different host molecules.

TTF (**1**) also forms inclusion complexes with neutral host molecules such as cyclodextrins (Figure 6). This complexation is mainly driven by the hydrophobic effect. α-Cyclodextrin (**6**) molecules encapsulate the hydrophobic TTF (**1**) in aqueous media [65]. Another water-soluble host which can complex TTFs is cucurbit[7]uril (**7**) [66]. However, it is not the neutral form, but the TTF radical cation **1**^●+^ which is preferably bound in the cavity of this host. Even the dication **1**^2+^ can be hosted by suitable macrocycles. For example, the π-electron-rich wheel **8**, consisting of two doubly-bridged 1,5-dioxynaphthalenes, is able to form a donor–acceptor complex with the π-electron-poor TTF^2+^ dication [26].

**Figure 7:**
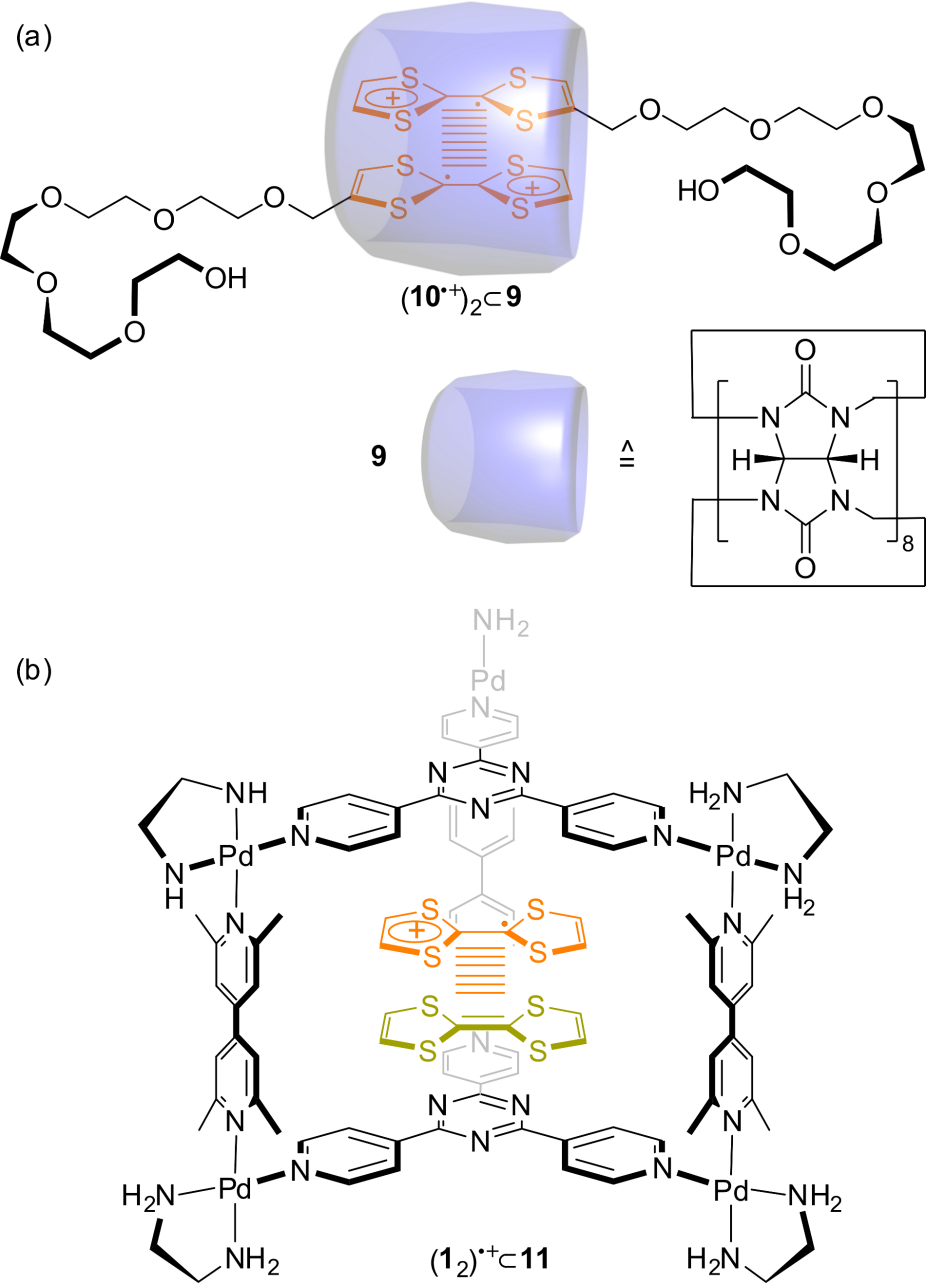
Stable TTF (a) radical-cation and (b) mixed-valence dimers in confined molecular spaces.

An astonishing discovery regarding TTF–cucurbituril complexes was made by Kim and co-workers in 2004 (Figure 7) [67]. The host molecule cucurbit[8]uril (**9**), which is enlarged by an additional glycoluril unit in comparison to **7**, provides sufficient space to accommodate two planar molecules with cofacial orientation [68]. When the water-soluble TTF derivative **10** gets oxidized to its radical cation **10**^●+^, a 2:1 complex is formed with a radical-cation dimer (**10**^●+^)_2_ stabilized in the cavity of the host molecule **9**. The presence of the radical-cation dimer complex (**10**^●+^)_2_
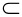
**9** was demonstrated by NMR, UV–vis and EPR spectroscopy. This was a novelty because stable TTF radical-cation dimers, which are usually only weakly associated species, where not characterized in aqueous medium at room temperature before.

A similar observation regarding TTF dimers was made by Fujita and co-workers in 2005 [69]. They used the self-assembled Pd-cage **11** to encapsulate two neutral TTF molecules. Oxidation of the solution yields an ambient-stable TTF mixed-valence dimer (**1**_2_)^●+^ inside the cage as shown by optical and electrochemical methods.

**Figure 8:**
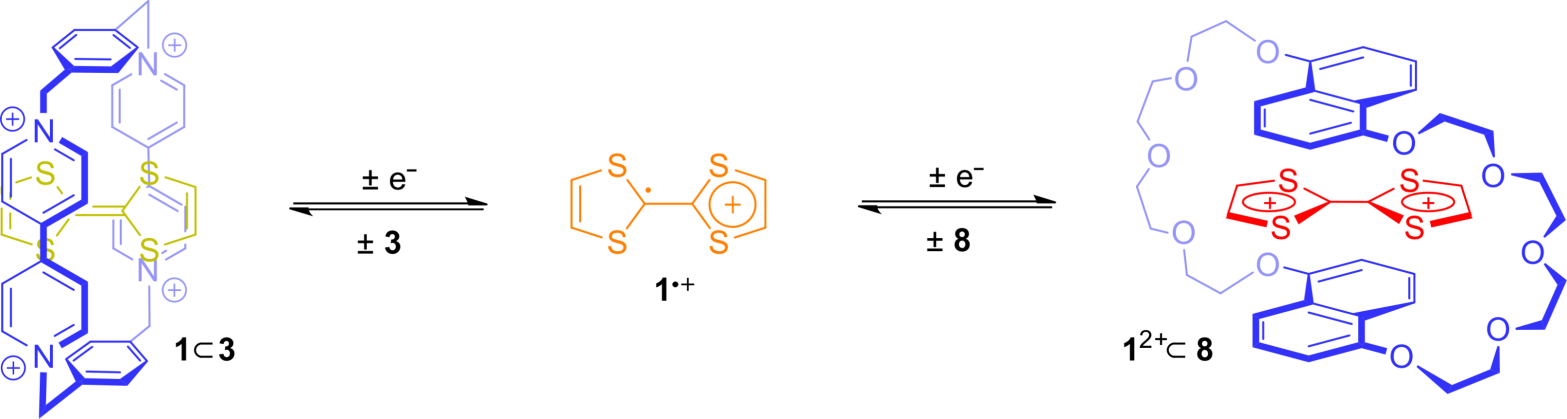
A “three-pole supramolecular switch”: Controlled by its oxidation state, TTF (**1**) jumps back and forth between different host molecules.

A further step towards motion control in MIMs was made by investigating the switching of the TTF molecule when different host molecules are available in solution (Figure 8). In a so-called “three-pole supramolecular switch” consisting of a mixture of host **3**, **8**, and TTF (**1**), the TTF molecule can change its position like in a “pea in the shell game” [26]. In its neutral form, TTF (**1**) forms the donor–acceptor complex **1**
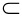
**3** with host **3**. Higher potentials need to be applied to oxidize TTF (**1**) into its radical-cationic form since the association energy of the donor–acceptor complex must be overcome. After oxidation, the radical-cation **1**^●+^ is expelled from host **3** by repulsive Coulombic forces. If **1**^●+^ gets further oxidized to the π-electron-poor dication **1**^2+^, the π-electron-rich macrocycle **8** can now encapsulate TTF^2+^. This relatively straightforward concept of electrochemically triggered complexation and expulsion of the TTF molecule from different hosts forms the fundament for motion control in a variety of different MIMs.

**Figure 9:**
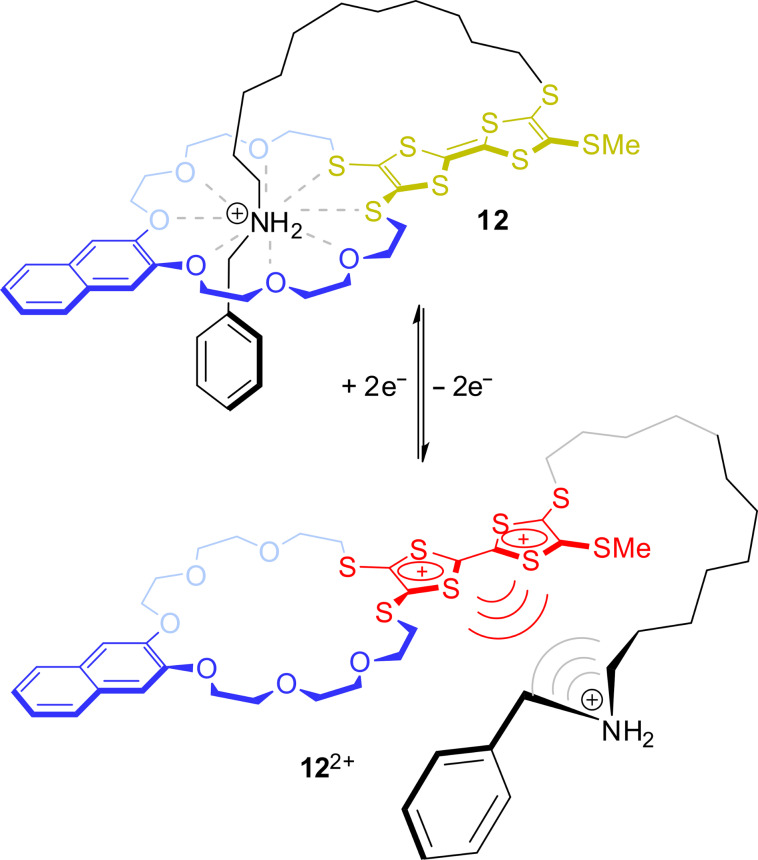
Redox-controlled closing and opening motion of the artificial molecular lasso **12**.

To illustrate how this redox-triggered complexation/decomplexation of pseudorotaxanes is transferred into a controlled molecular motion in MIMs, the TTF-based pseudo[1]rotaxane **12** recently reported by us is shown in Figure 9 [70]. In a pseudo[1]rotaxane, the axle molecule is covalently bound to the wheel component. The self-inclusion structure mimics the conformation of a molecular lasso, a structural motif which was also recently found in nature for peptides with high antibacterial efficacy [71]. In **12**, the TTF molecule is not implemented in the thread but in the wheel component. In the neutral state, strong hydrogen bonding between the crown ether wheel and the dialkylammonium station forces a threaded conformation. If the TTF is oxidized, however, charge repulsion between the ammonium station and the TTF moiety weakens the binding. This ultimately leads to an expulsion of the thread from the cavity of the wheel, and thus to an opening of the lasso. This example shows how easily a reversible molecular motion can be achieved by redox-switching of the TTF unit.

### 4. Rotaxanes

Rotaxanes consist of a dumbbell-shaped axle molecule encircled by a macrocycle. Bulky stopper groups at both ends of the axle prevent a deslipping of the wheel. With a development starting from low-yielding statistical synthesis to efficient template-controlled synthesis, rotaxanes have become the workhorse of MIMs in the last three decades. Regarding their use as molecular devices, three types of motions can be potentially controlled in rotaxanes: pirouetting of the wheel around the axle, translation of the wheel along the axle, and a rocking motion of the wheel [72]. However, most reports have focused on the stimuli-controlled translational motion. MIMs in which the wheel position on the axle is controlled by external stimuli are called “switchable molecular shuttles”.

#### 4.1. Switchable molecular shuttles

**Figure 10:**
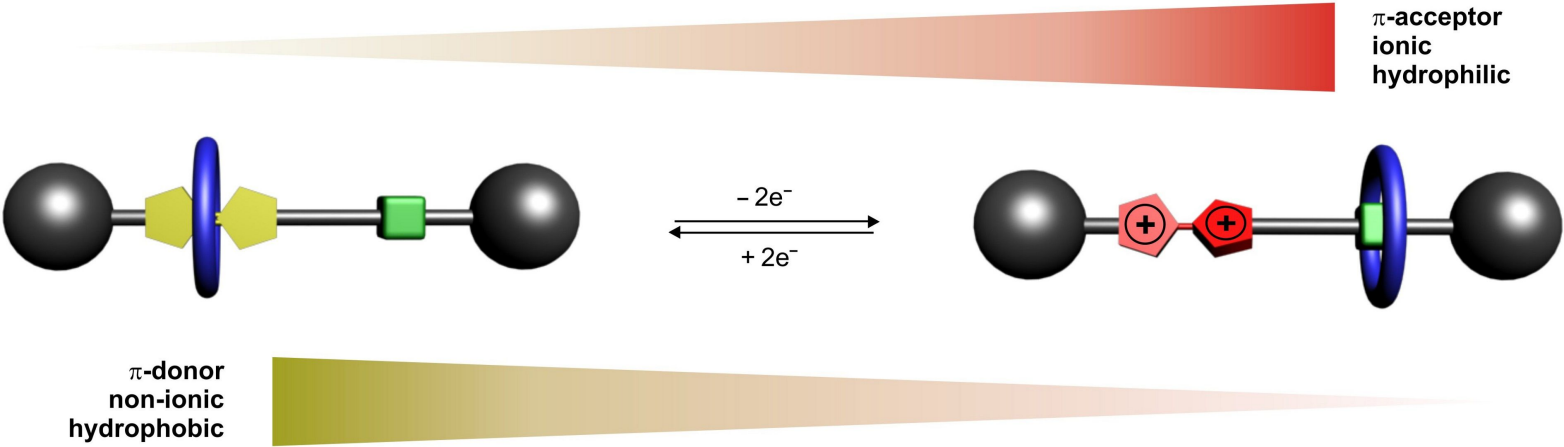
Graphical illustration how a non-degenerate TTF-based shuttle works under electrochemical operation.

A schematic illustration of the working principle of non-degenerate TTF-based shuttle rotaxanes is shown in Figure 10. In the ground state co-conformation, the wheel encircles the TTF unit. Upon TTF oxidation, the attractive forces are lowered or even repulsive forces are generated between the TTF unit and the wheel inducing a motion of the wheel towards the now energetically preferred green-colored binding site. Therefore, the most populated and consequently the ground-state co-conformation in the oxidized state is the wheel on the green station. Because of the reversibility of TTF redox reactions, the molecular shuttle can be reversibly switched over many cycles. The work which is generated in the operation of a molecular shuttle is reminiscent of a piston engine used for macroscopic motors. However, one should keep in mind that the transfer of the concepts of macroscopic machines to the molecular level may be limited and may even be misleading. In contrast to macroscopic piston engines, the translation of the wheel in a rotaxane occurs through Brownian motion and the switching processes cause merely a shift of the equilibrium between the two positional isomers of the rotaxane. Thus, a transfer of work on the molecular level that is created by wheel translation into a macroscopic force is quite difficult (but not impossible) to achieve.

**Figure 11:**
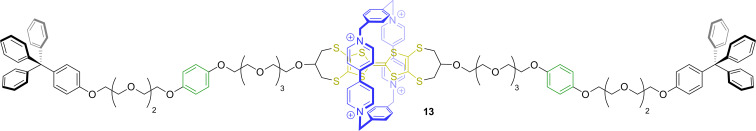
The first TTF-based rotaxane **13**.

Closely after the discovery of the donor–acceptor complex **1**
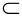
**3**, Stoddart and co-workers reported the synthesis of the first TTF-based rotaxane **13** (Figure 11) [73]. The [2]rotaxane was obtained in 8% yield by a high-pressure clipping procedure in which the wheel **3** was formed around the pre-synthesized axle. In DMSO, the macrocycle is predominantly located on the central TTF moiety. However, in acetone, which has a lower polarity, the wheel moves to one of the dihydroxybenzene residues as indicated by ^1^H NMR and UV–vis spectroscopy. Since the axle molecule is symmetric, the wheel moves back and forward between the two dihydroxybenzene stations (green) and the rotaxane can be considered as a degenerate shuttle.

**Figure 12:**
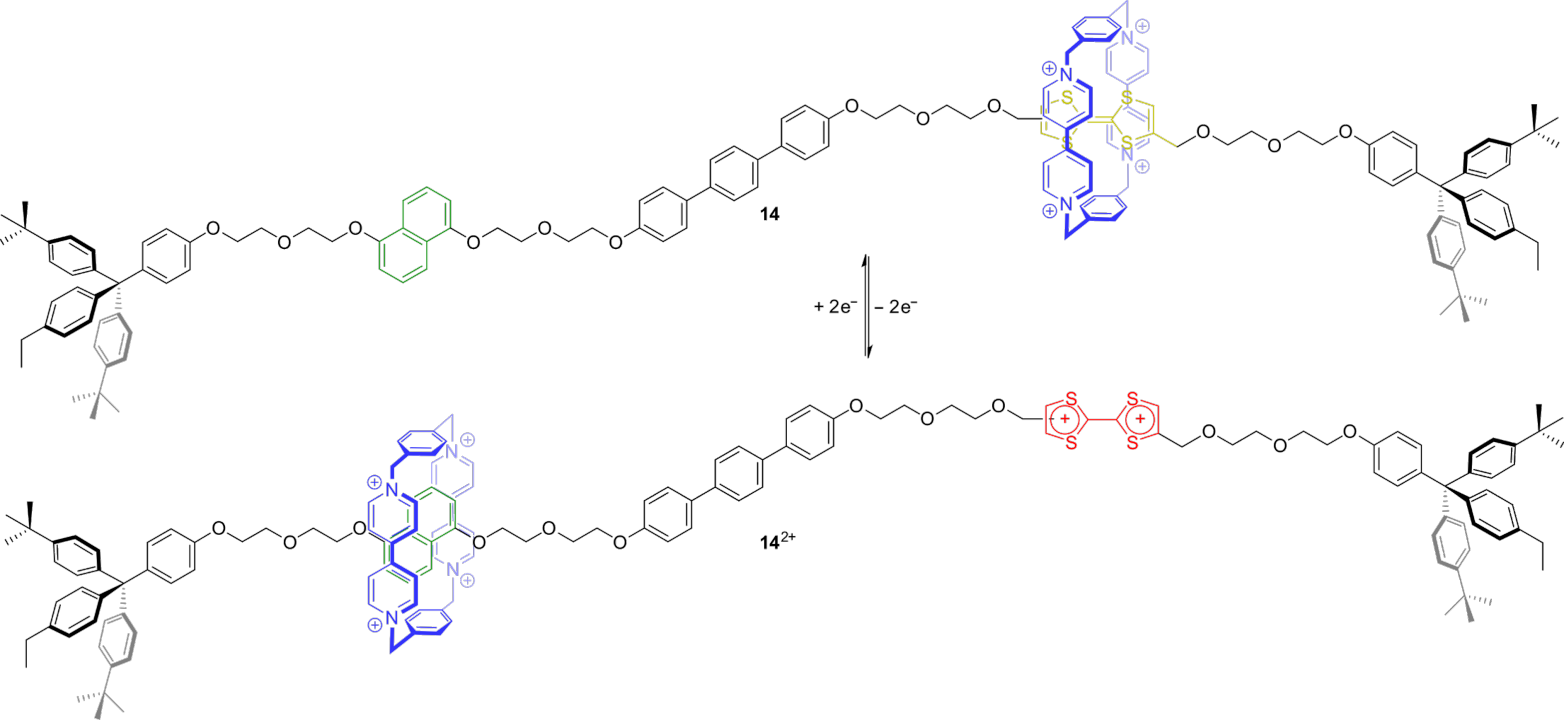
A redox-switchable bistable molecular shuttle **14**.

Subsequently, the groups of Becher and Stoddart reported on a series of similar, but non-degenerate [2]rotaxanes [74–75]. After several structural optimizations, the bistable rotaxane **14** with a high switching efficiency was reported in 2003 (Figure 12) [76]. In the unswitched state, host **3** is located at the TTF binding site. Chemical oxidation to the dication TTF^2+^ triggers a translational motion of the wheel towards the 1,5-dihydroxynaphthalene station (green) as shown by UV–vis and 2D NMR experiments. Chemical reduction with zinc powder restored the spectroscopic properties of the starting state and back-shuttling of the wheel to the TTF station occurs. In the TTF^2+^ state, no signals for a free 1,5-dihydroxynaphthalene station were observed which indicates the rotaxane to be completely switched within the detection limit of ^1^H NMR spectroscopy. The experimental observations and the general switching mechanism of these bistable donor–acceptor rotaxanes were subsequently underpinned by several quantum mechanical studies [77–79].

**Figure 13:**
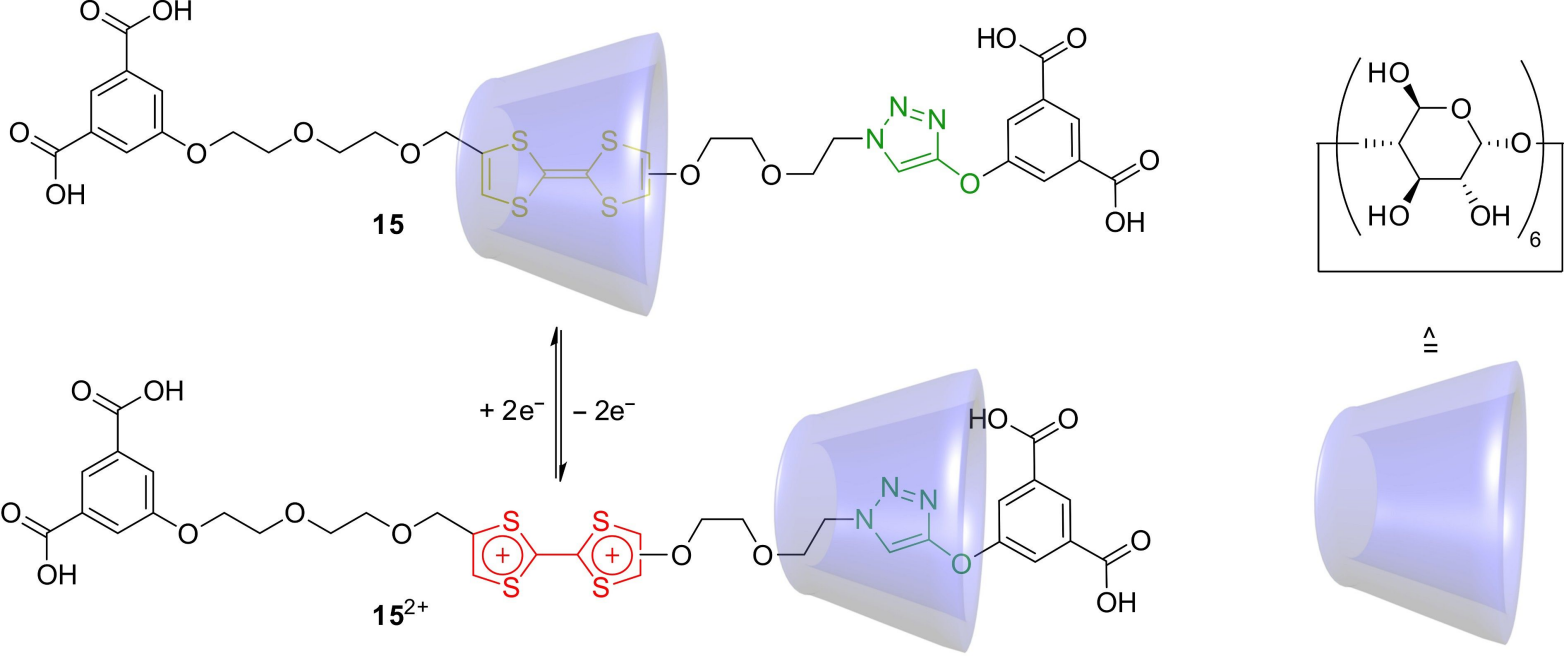
The redox-switchable cyclodextrin-based rotaxane **15**.

The concept of TTF-based switchable rotaxanes was also extended to rotaxanes with other macrocycles. In Figure 13, the bistable [2]rotaxane **15** is shown with an α-cyclodextrin (**6**) ring threaded onto a water-soluble axle [80]. The rotaxane was synthesized in 23% yield by a template/capping strategy where one stopper is attached using copper-catalyzed azide–alkyne click chemistry after the formation of the precursor pseudorotaxane. Due to the hydrophobic effect, the neutral TTF is preferred as a station for the wheel over the triazole unit. However, after oxidation, the more hydrophilic dication TTF^2+^ is less favored and the ring moves to the triazole. Both switching states were fully characterized by UV–vis, ^1^H,^1^H-NOESY NMR spectroscopy, and cyclic voltammetry. The latter technique revealed an increase of oxidation potential for the first one-electron oxidation, but a second oxidation potential similar to that of the free axle. This indicates that the wheel already moves away from the TTF station upon the first oxidation to the TTF^●+^ radical cation.

**Figure 14:**
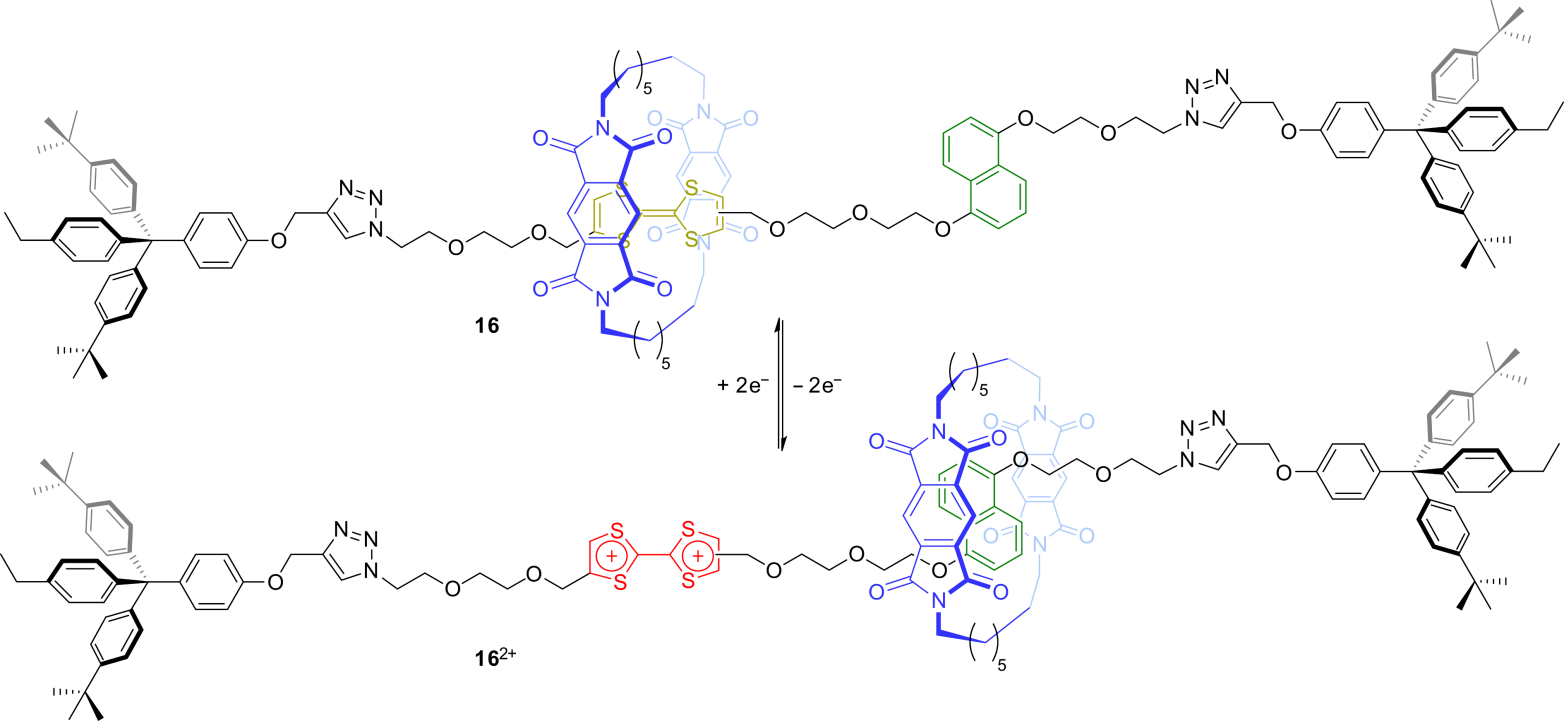
The redox-switchable non-ionic rotaxane **16** with a pyromellitic diimide macrocycle.

Another example for a non-charged TTF-based rotaxane was reported in 2011 (Figure 14) [81]. Very similar to the prior bistable rotaxanes, donor–acceptor interactions dominate the relation of wheel and axle in [2]rotaxane **16**. Here, a π-electron-deficient pyromellitic diimide macrocycle encircles a TTF station (*K*_a_ = 6,300 M^−1^) which is embedded in an axle molecule with two azide residues. The second station, the dihydroxynaphthalene moiety (green), displays a lower association constant of *K*_a_ = 5,800 M^−1^. The pseudorotaxane precursor was end-capped by a double copper-catalyzed azide–alkyne click reaction in CH_2_Cl_2_ and the rotaxane was isolated in 34% yield. NMR spectroscopy, cyclic voltammetry, and spectroelectrochemistry showed that the wheel translates to the dihydroxynaphthalene station upon TTF oxidation.

**Figure 15:**
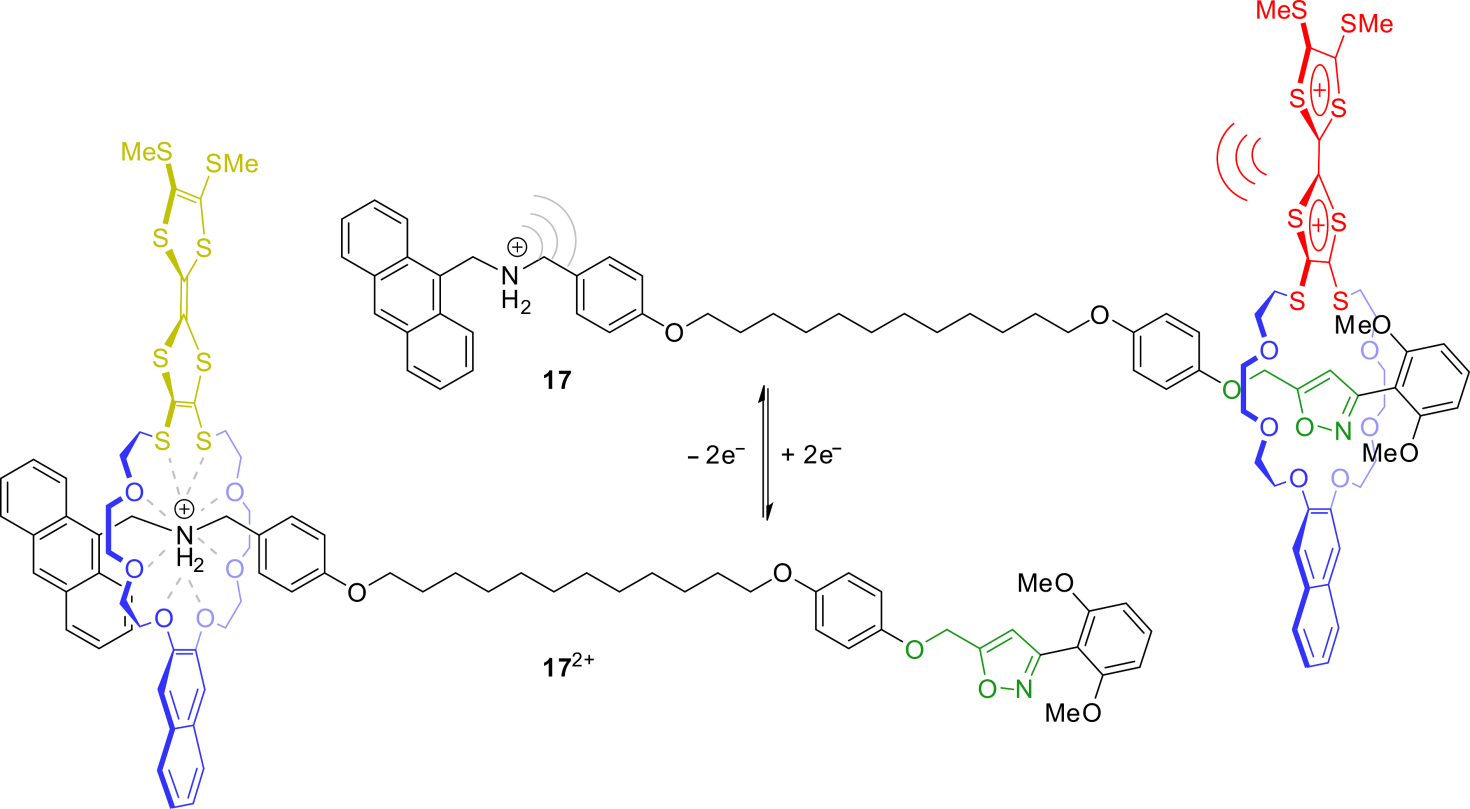
The redox-switchable TTF rotaxane **17** based on a crown/ammonium binding motif.

The use of TTF in redox-switchable rotaxanes is not limited to the implementation into axle components. Very recently, our group reported on a crown/ammonium rotaxane **17** in which a TTF unit is implemented in the crown-ether wheel (Figure 15) [82]. The rotaxane was synthesized by a catalyst-free nitrile oxide capping strategy in 67% yield. In the neutral state, the wheel is strongly bound to the ammonium station by hydrogen bonds as shown by the high association constant of a structurally similar pseudorotaxane precursor (*K*_a_ = 590,000 M^−1^). The high association constant is a result of the weakly coordinating anion (WCA) used, i.e., tetrakis(3,5-bis(trifluoromethyl)phenyl)borate. Comparison to a structurally similar rotaxane in which the ammonium station is blocked by *N*-acetylation shows that the isoxazole moiety acts as a weak second binding station for the wheel. Oxidation of the TTF unit results in Coulombic repulsion between the wheel and the ammonium station which counteracts the energy of hydrogen bonding. Detailed electrochemical measurements and digital simulations revealed the ring still to be bound to the ammonium station in the TTF^●+^ state. However, after double oxidation a wheel distribution of 1:1 between the ammonium and the isoxazole station was found indicating a dynamic motion between both stations.

#### 4.2. Optical devices

The rapid development of redox-switchable MIMs led to considerations to use the unique properties of these molecules for different optoelectronic devices. TTF-based MIMs often display very long-living and stable switching states which are particularly appealing for applications as data storage devices [83] or molecular logic gates [84]. Besides simple molecular electrochromic switches, complex TTF-based MIMs with unique optical properties have been constructed.

**Figure 16:**
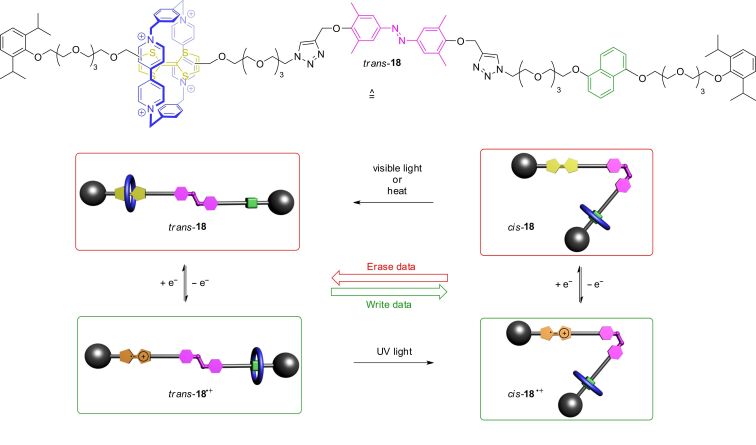
Structure and operation of the electro- and photochemically switchable rotaxane **18** which acts as potential memory device.

In 2012, rotaxane **18** with a structure similar to previously reported donor–acceptor rotaxanes was reported (Figure 16) [85]. However, the axle bears a central azobenzene photoswitch, who’s *E*/*Z*-transitions can be controlled by light. The redox-switchable shuttle works as previously described and the position of the wheel can be controlled by oxidation or reduction. However, the configuration of the azobenzene strongly influences the life time of each redox-switching state. In the (*E*)-form, the wheel can easily move between the TTF and dihydroxynaphthalene station. In the *Z*-form, this movement is sterically hindered and slowed down. The wheel cannot shuttle to its energetically preferred station. This possibility of orthogonal switching (redox and light) enables an electrochemical “writing” of data which can be subsequently locked by a light stimulus.

**Figure 17:**
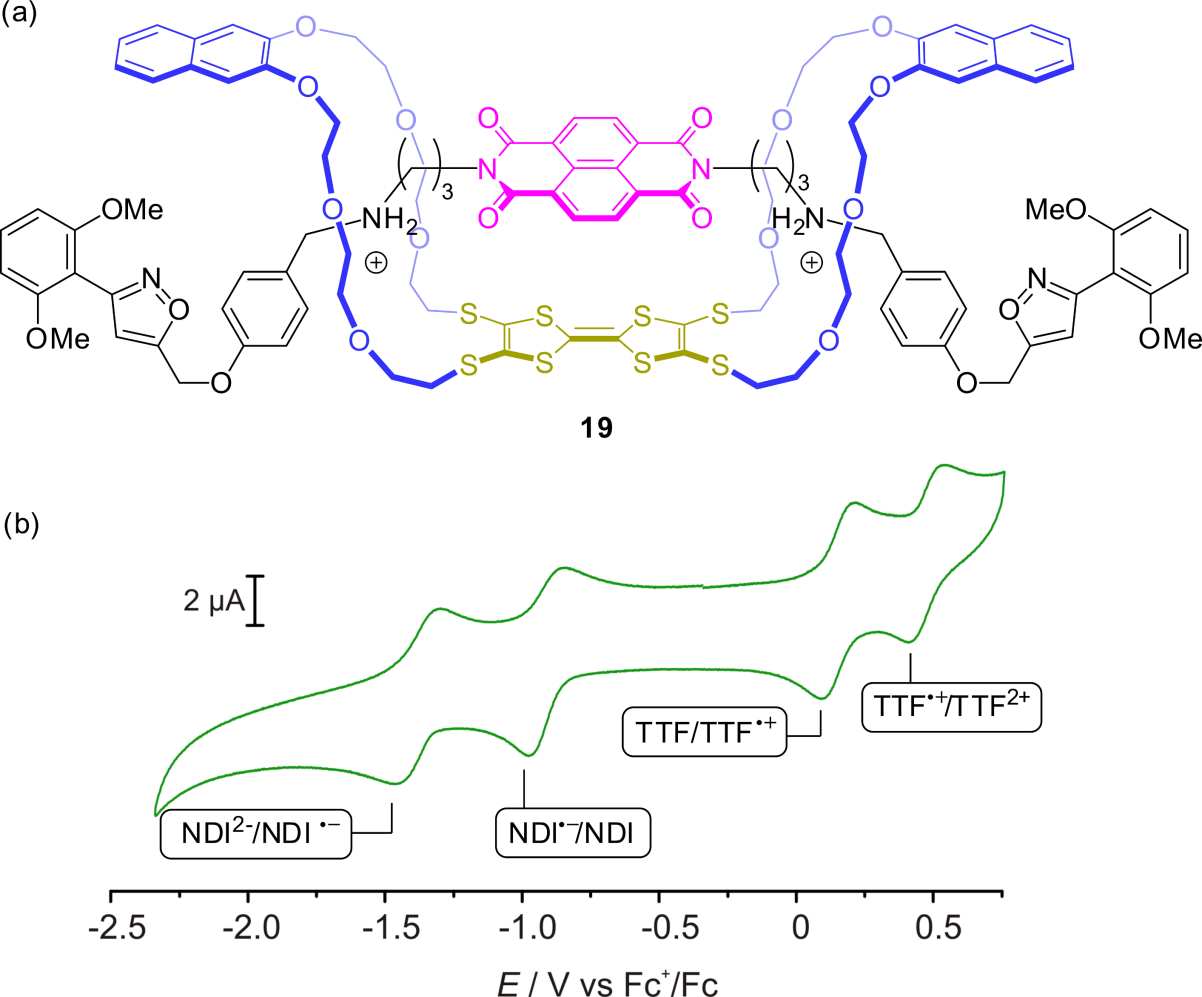
(a) The redox-switchable rotaxane **19** with a donor–acceptor pair which is stable in five different switching states. (b) Cyclic voltammogram showing the transitions between the five oxidation states of **19**.

The TTF-based doubly interlocked crown/ammonium rotaxane **19** recently reported by us consists of a divalent axle with a π-electron-poor central naphthalene diimide (NDI) unit and a divalent crown-ether wheel with a central TTF unit (Figure 17) [86]. The cofacial donor–acceptor complex whose formation is indicated by a deep green color is forced to stay in a cofacial orientation by mechanical bonding, even when the complex is redox-switched. UV–vis spectroscopy showed the emergent charge-transfer absorption band to display a negative solvochromic effect. Similar to TTF, the NDI unit has three stable oxidation states. However, these are neutral or anionic states and successive reduction of NDI leads to the radical anion and the dianion. Cyclic voltammetry, DFT calculations, and UV–vis spectroscopy confirmed five different redox states (TTF/NDI, TTF^●+^/NDI, TTF^2+^/NDI, TTF/NDI^●−^, TTF/NDI^2−^) and shows interesting optical properties in each of these redox states making this type of mechanically constrained donor–acceptor complex very interesting for molecular electronic materials and optoelectronic devices.

#### 4.3. TTF-Based rotaxanes on solid support

If one aims at creating macroscopic effects, the concerted action of many molecular machines is needed. It is then useful to deposit switchable AMMs on interfaces such as a surface of a solid support [87–89]. An ordered array of molecules enables the possibility of concerted switching. The fixed orientation on a surface allows studying molecules with sophisticated techniques such as scanning tunneling microscopy. Furthermore, redox-switchable AMMs, containing for example TTF moieties, can be electrically operated without the need of chemical additives, if conducting solid supports are used. This also opens pathways towards the integration of switchable AMMs into the world of silicon-based chips and electronic circuits.

**Figure 18:**
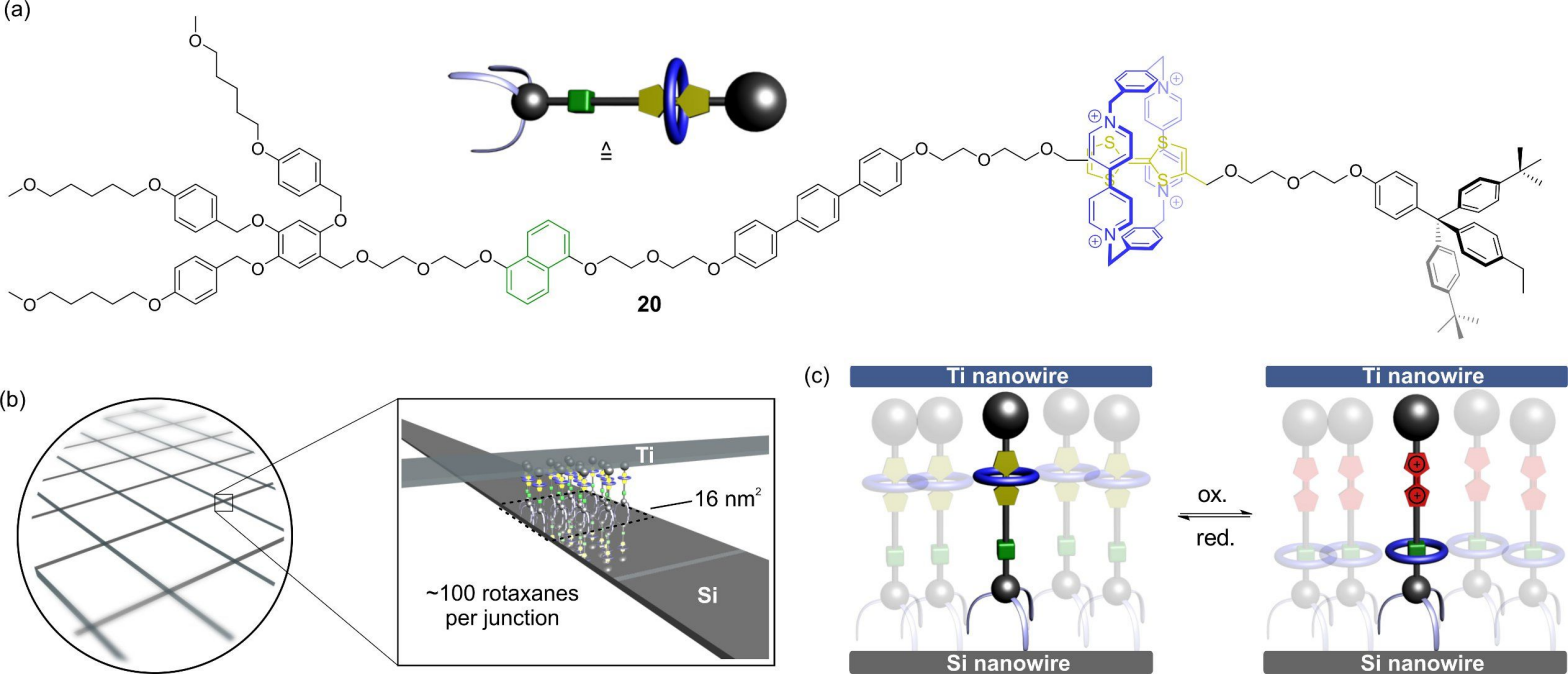
Schematic representation of a molecular electronic memory based on a bistable TTF-based rotaxane. (a) Molecular structure of the amphiphilic [2]rotaxane **20**. (b) Structure of the crossbar device. (c) Switching mechanism of rotaxane **20** in a junction.

A landmark in the field of molecular-scale electronic devices is the rotaxane-based 160-kilobit memory which was reported by the groups of Stoddart and Heath in 2007 (Figure 18) [90]. The key idea of this memory is that the switching modes in a bistable rotaxane **20** can be considered as the “1” and “0” states of a binary digit. If the rotaxane shows a hysteretic current–voltage curve, a voltage-induced reading and writing of information becomes possible. Although the initial concept of this type of device was developed a few years earlier [91], the optimization of its structure and fabrication was necessary to reach high-level performance [92–94].

The device is based on a crossbar architecture in which a monolayer of amphiphilic rotaxanes is sandwiched by a bottom Si nanowire electrode and a top Ti nanowire electrode. Both layers of nanowires are orthogonal to each other. This produces several crossing points or “junctions” whose areas are defined by the diameter of the nanowires. In the present example, a very small diameter gives a junction area of only 16 nm^2^ which corresponds to approximately 100 rotaxanes per junction. When a high pulse voltage is applied (±1.5 V), the junctions can be switched back and forward between the two switching states of the rotaxane. The written information is then read out by a non-perturbing lower voltage. It turned out that the terphenyl spacer which is implemented into the rotaxane axle is crucial to increase the half-life of the metastable switching state. However, a disadvantage of this particular device was a high fraction of “defect” junctions. Only ≈25% showed a sufficient on/off ratio for a memory device. Furthermore, only a limited number of switching cycles was possible before the junctions were damaged. However, a remarkably high storage density of 10^11^ bits cm^−1^ was reached.

Writing of data with the aid of dendrimer-decorated TTF-rotaxanes was achieved by Gao and co-workers [95]. They deposited a thin-film of bistable donor–acceptor rotaxanes on an indium tin oxide (ITO)-coated substrate. A clean electrochemical switching on the substrate was observed with current–voltage curves showing a clear memory effect. The written data could be read out even after waiting for 12 h.

**Figure 19:**
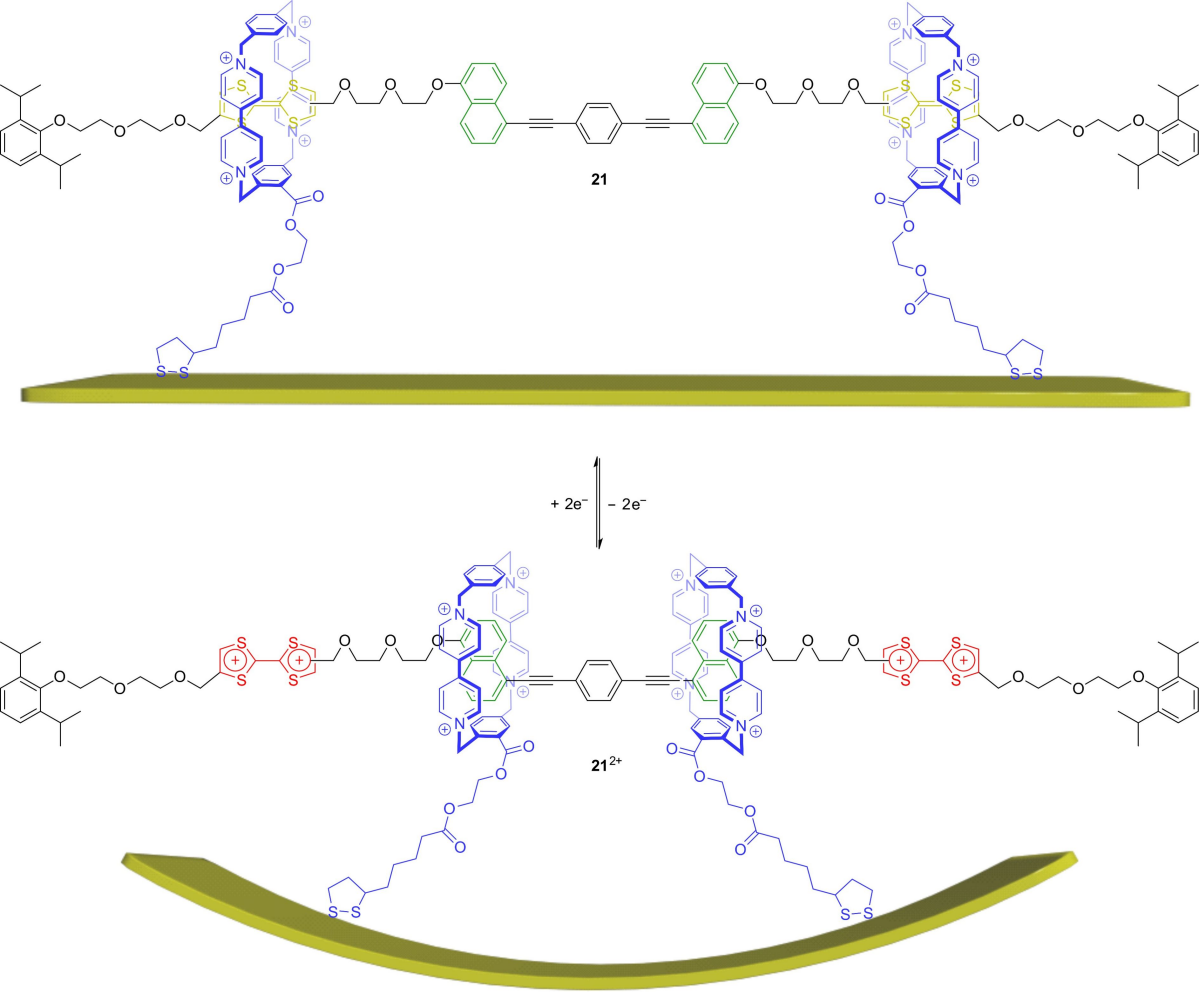
Schematic representation of bending motion of a microcantilever beam with gold surface induced by operation of the redox-switchable [3]rotaxane **21** attached to its surface.

Besides data storage, a substantial challenge of AMMs is the transfer of molecular motion into a useful macroscopic output. An example of rotaxanes on a solid support which could achieve this is shown in Figure 19 [96]. The [3]rotaxane **21** consists of a symmetric axle molecule in which both axle halves bear a TTF and a hydroxynaphthalene station. In the unswitched state, each TTF station is encircled by a wheel. Oxidation of the TTF units then induces shuttling motions towards the inner hydroxynaphthalene stations, which significantly reduces the wheel–wheel distance. If both wheels are attached to a surface with a suitable anchor, the shuttle motion can be seen as a type of muscle-like contraction generating tensile stress on the surface. Although the force of one contracting rotaxane is quite small, a sufficient number of these “molecular muscles” can accumulate their force and consequently deform a material by concerted switching.

In rotaxane **21**, the two macrocycles are attached by disulfide anchors to the gold surface of microcantilever beams (500 × 100 × 1 µm) and form a self-assembled monolayer. Chemical oxidation leads to a bending and to an upward motion of the beams by ≈35 nm. The addition of ascorbic acid as reductant restores the initial position and the switching cycle can be repeated. To exclude other triggers than the rotaxane contraction, several control experiments were performed. Furthermore, a structurally similar but mechanically inert control compound was synthesized which cannot induce the bending effect.

#### 4.4. TTF Pairing interactions in rotaxanes

**Figure 20:**
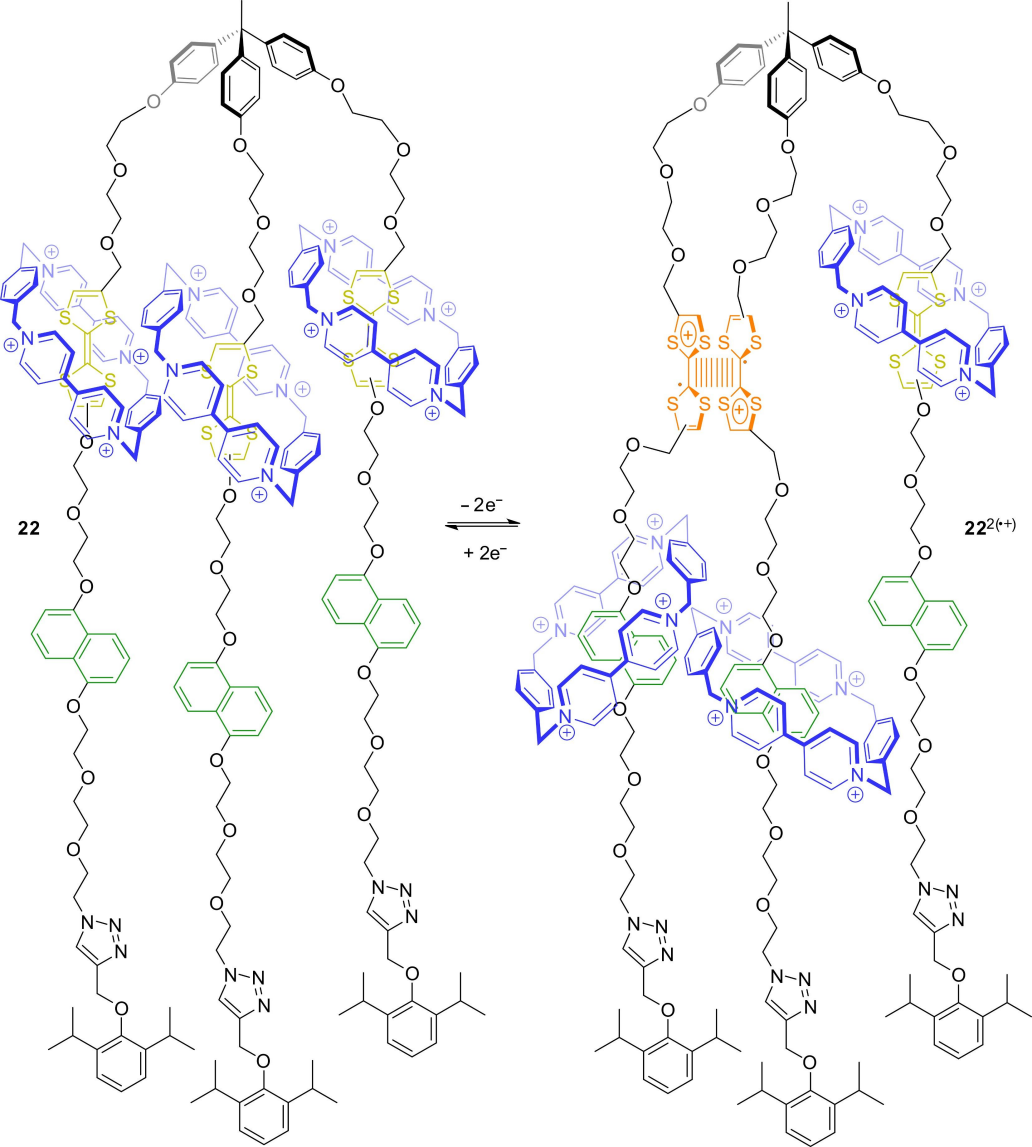
TTF-dimer interactions in a redox-switchable tripodal [4]rotaxane **22**.

Although TTF is widely used in switchable molecular shuttles, rotaxanes with TTF–dimer interactions are rare. One example was published by Stoddart and co-workers in 2008 (Figure 20) [97]. The tripodal [4]rotaxane **22**, consisting of a trivalent axle, in which each arm is encircled by a host molecule **3**, was synthesized by a copper-catalyzed click protocol in 40% yield. A combination of electrochemical and spectroscopic methods was used to investigate a potential TTF-dimer formation. As comparison, they characterized also the trivalent dumbbell precursor without wheels. It was shown that mixed-valence interactions (TTF_2_)^●+^ and radical-cation interactions (TTF^●+^)_2_ are present during the successive oxidation of the free axle. However, only the radical-cation dimer interaction was observed in the case of [4]rotaxane **22**. This discrepancy can be explained by a simple energy balance. For a mixed-valence interaction, at least two TTF/TTF^●+^ units need to be free. Thus, after one-electron oxidation which liberates one TTF^●+^ from the cavity of **3**, still the energy of one donor–acceptor complex has to be overcome to enable a mixed-valence interaction. This is not the case for the free trivalent axle. After double oxidation, two TTF^●+^ stations are free and a radical-cation interaction is favored. This example nicely shows that all energy contributions in each switching state of a system as a whole need to be considered for the design and operation of an AMM.

**Figure 21:**
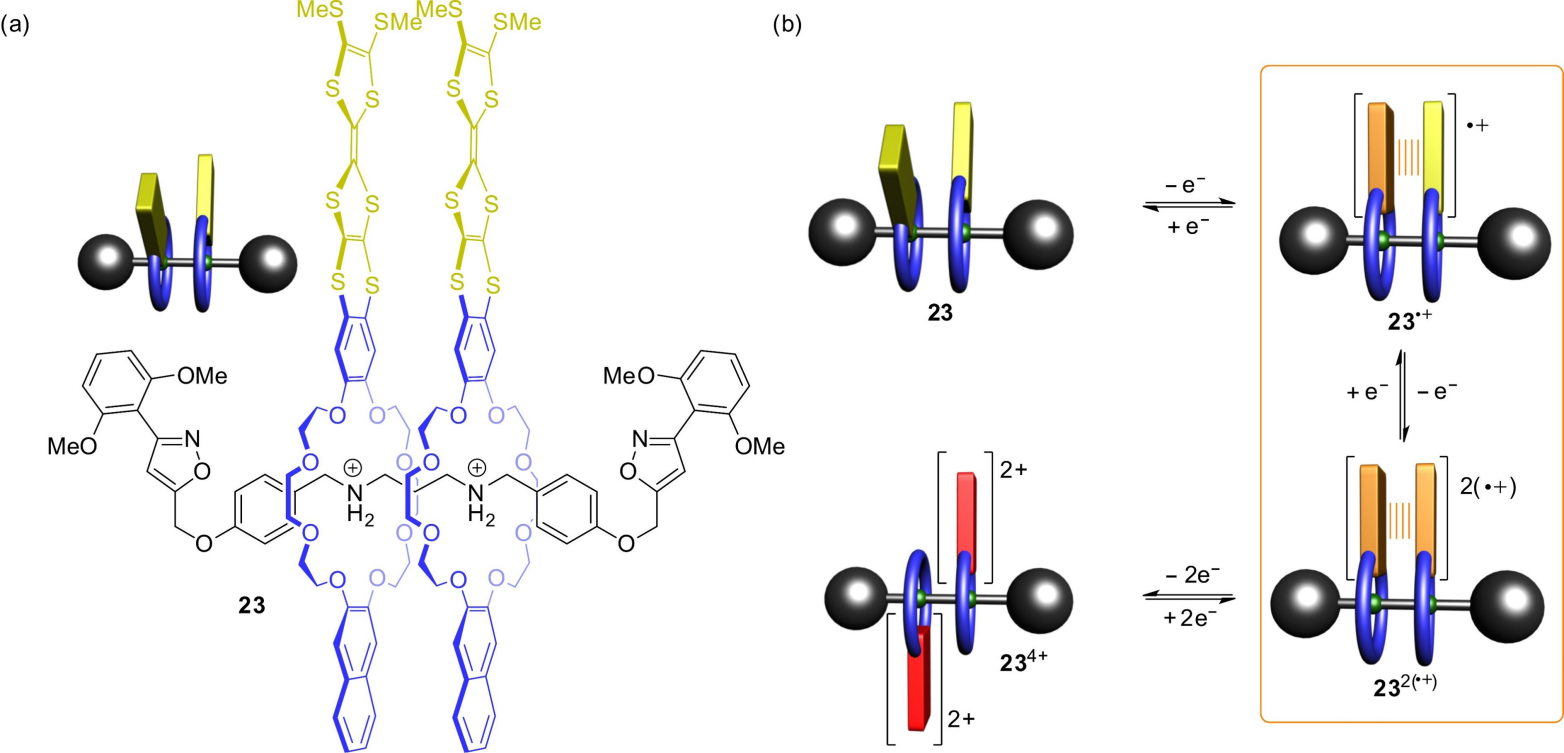
(a) A molecular friction clutch **23** which can be operated by electrochemical stimuli. (b) Schematic representation of **23** in its four stable oxidation states with corresponding wheel co-conformations.

In addition to the variety of TTF-based rotaxane shuttles, we recently reported a [3]rotaxane in which the pirouetting motion of wheels can be controlled by electrochemical switching [98]. Figure 21 shows the [3]rotaxane **23** which bears two cofacially oriented TTF crown ethers on a divalent ammonium axle. The distance between the wheels is convenient for TTF-dimer interactions. In the non-switched neutral state of both TTFs, the wheels adopt a *syn* co-conformation caused by weak non-covalent interactions between the wheels. One-electron oxidation yielding **23**^●+^ enables mixed-valence interactions (TTF_2_)^●+^ between the cofacial TTF units. This TTF-dimer interaction “clutches” the two wheels and synchronizes their pirouetting motions around the axle. Also the next stable oxidation state (**23**^2(●+)^) shows attractive wheel–wheel interactions in form of a TTF radical-cation dimerization (TTF^●+^)_2_. However, further oxidation leads to the fully oxidized **23**^4+^ in which both TTF^2+^ units repeal each other. The Coulombic repulsion “declutches” the two wheels and they adopt an *anti* co-conformation. As shown by experiments and quantum chemical calculations, the wheels cannot be fully disengaged; however, the wheel–wheel interactions strongly differ for the different oxidation states. The controlled clutching and declutching of **23** by electrochemical stimuli is reminiscent of the operation of a macroscopic friction clutch, a common technical device used in motor vehicles. Furthermore, rotaxane **23** can be used as novel supramolecular gearing system for the transmission of rotational motion at the molecular level.

### 5. Rotacatenanes

**Figure 22:**
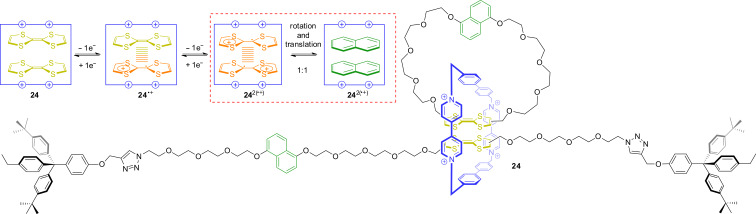
Fusion between rotaxane and catenane: a [3]rotacatenane **24** which can stabilize TTF dimers.

In 2011, the group of Stoddart described the fusion of a rotaxane and a catenane, a so-called “rotacatenane” (Figure 22) [99]. The rotacatenane **24** consists of their previously used rotaxane framework except that the enlarged cyclophane cyclobis(paraquat-4,4′-biphenylene) is used as wheel component. The cavity of this macrocycle is large enough to host two planar molecules in a cofacial arrangement. Starting from the pre-assembled catenane, the axle molecule is threaded through the wheel and end-capped by a copper-catalyzed click reaction.

A variety of different spectroscopic and electrochemical methods was applied to reveal the switching behavior of **24** and its stable co-conformations in each switching state. In the unswitched state, both TTF units are stacked in the cavity of the wheel. One-electron oxidation to **24**^●+^ creates a stabilized mixed-valence interaction between these units as evidenced by an emergent NIR band. Further oxidation, converts the two TTF units into a radical-cation dimer (TTF^●+^)_2_. However, the Coulombic repulsion in this six-fold charged complex destabilizes the radical-cation dimer and the system converts into a second stable co-conformation in which both dihydroxynaphthalene units are inside the cavity of the wheel. To achieve this conformer, two types of motion, a circumrotation and a translational motion, must occur. The equilibrium between these two co-conformations of **24**^2(●+)^ was determined to be approximately 1:1. Further oxidation drives the equilibrium completely to the side of the co-conformation in which both dihydroxynaphthalene units are encircled. This TTF-based system is an intriguing example of synergetic molecular motions triggered by redox stimuli.

### 6. Catenanes

Catenanes consist of at least two intertwined macrocycles which are mechanically interlocked. The structure cannot be opened without breaking a covalent bond. In contrast to rotaxanes in which the wheel is only held on the axle component by steric hindrance of stopper groups, a catenane is a truly topologically interlocked species bearing a mechanical bond. However, the construction, chemical behavior, and operation of structurally related rotaxanes and catenanes are often very similar. The motion which can be controlled by external stimuli is the rotation (or circumrotation) of the wheels relative to each other.

**Figure 23:**
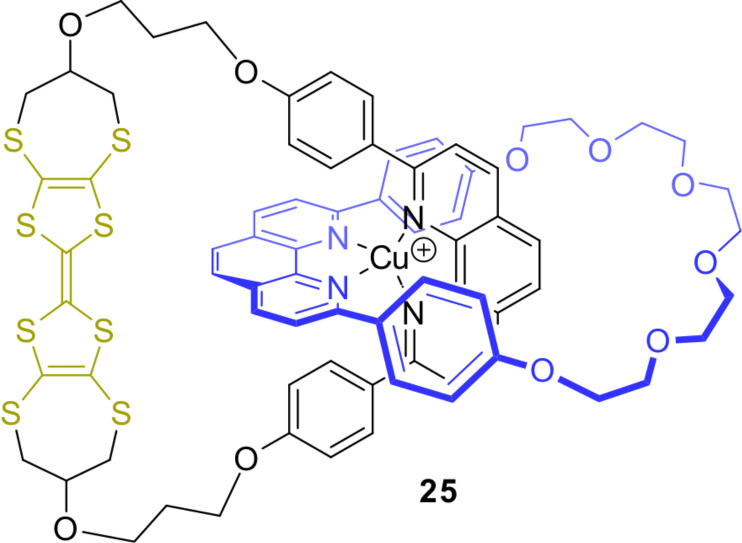
The first TTF-based catenane **25**.

The first TTF-based catenane **25** was described by the groups of Becher and Sauvage in 1994 (Figure 23) [100]. Starting from a phenanthroline macrocycle bearing a TTF unit, a copper(I)-template was used to obtain the TTF-based catenane **25** in 14% yield, which was previously developed by Sauvage [101]. The authors aimed for a further development of this construction motif towards donor–acceptor rotaxanes with efficient charge separation leading to a broad variety of topologically complex TTF catenanes and cage compounds [52,74,102–104].

#### 6.1. Stimuli-responsive circumrotation

**Figure 24:**
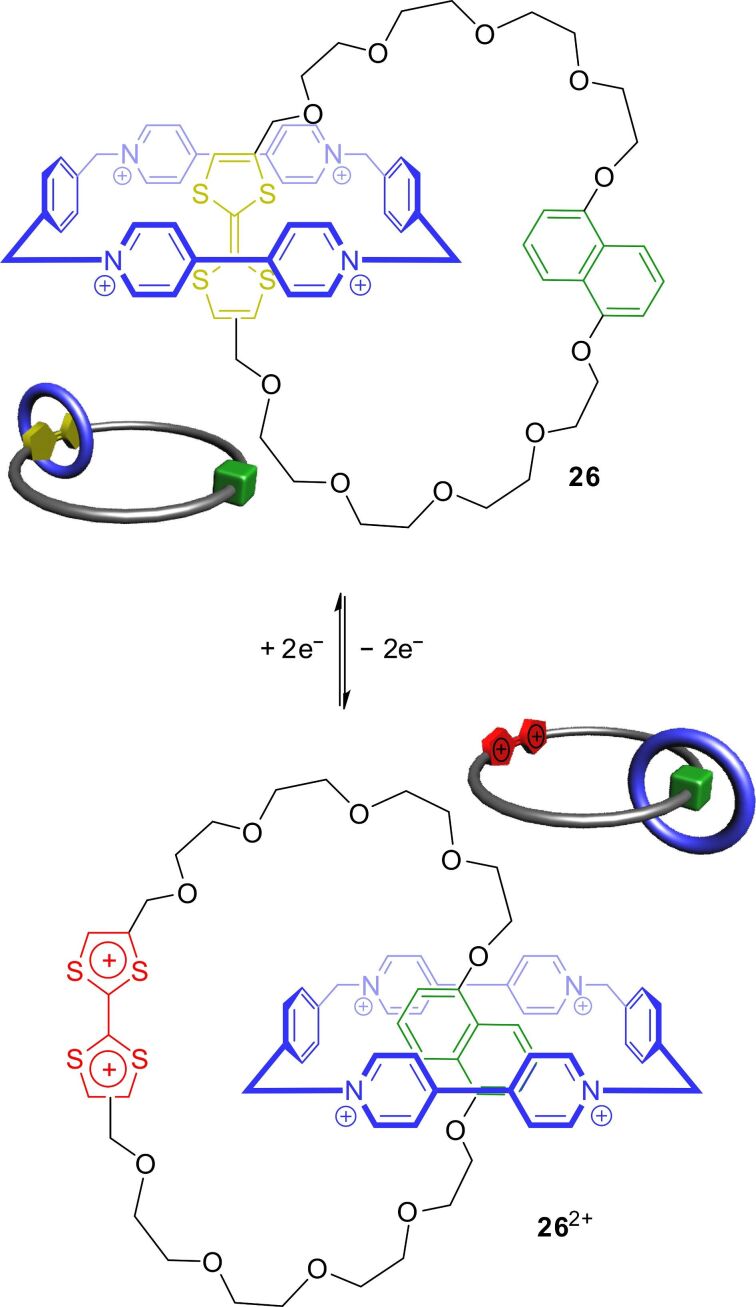
Electrochemically controlled circumrotation of the bistable catenane **26**.

The TTF-based catenane **26** allows implementing a stimuli-responsive circumrotation motion (Figure 24) [105]. Similar to the corresponding donor–acceptor rotaxanes, the macrocycle preferentially encircles the TTF unit instead of the dihydroxynaphthalene station in the unswitched state. As shown by ^1^H NMR and UV–vis spectroscopy as well as cyclic voltammetry, chemical oxidation to the TTF^2+^ dication triggers an expulsion of the former station and the wheel moves to the alternative dihydroxynaphthalene station. Chemical reduction with ascorbic acid or Na_2_S_2_O_5_ restores the initial spectroscopic properties and the initial co-conformation of the catenane.

**Figure 25:**
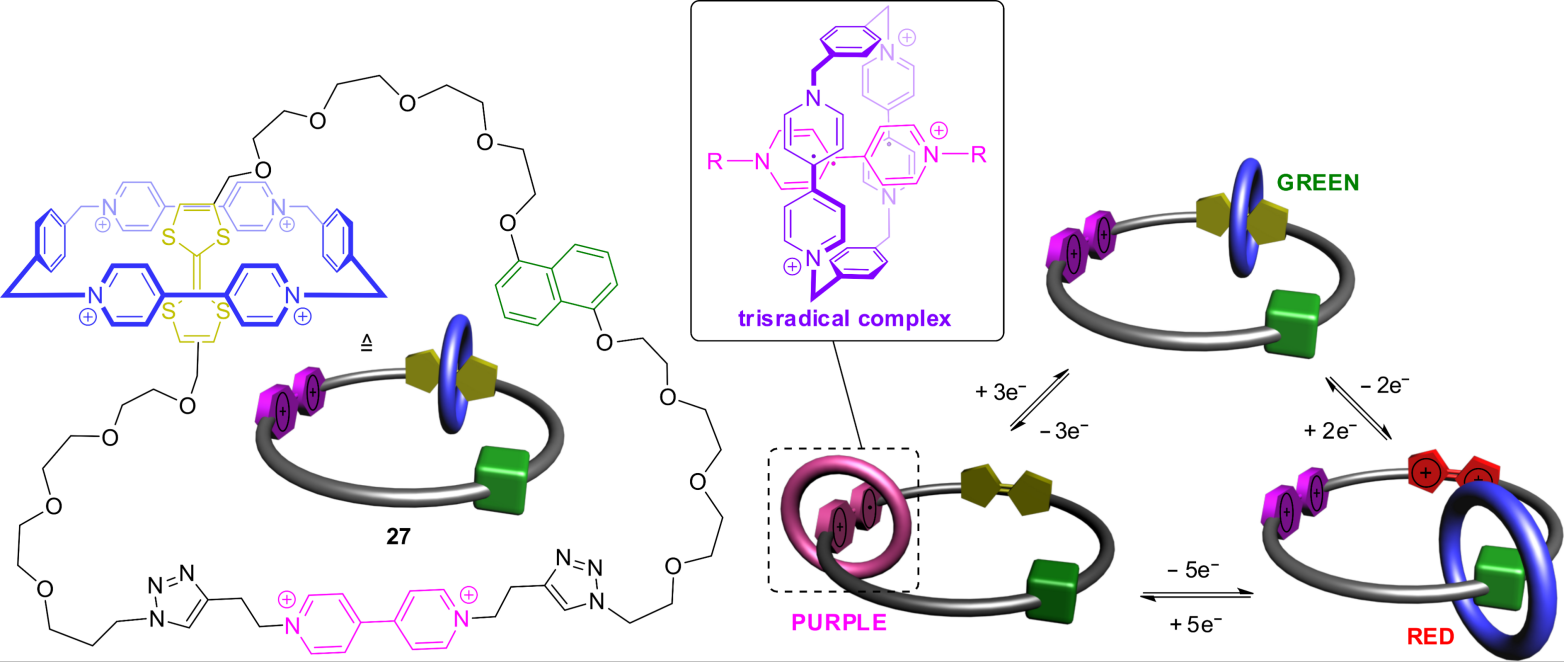
A tristable switch based on the redox-active [2]catenane **27** with three different stations.

A tristable molecular switch based on a [2]catenane with three different stations was created by Wasielewski, Stoddart, and co-workers in 2015 (Figure 25) [106]. The catenane **27** is made of a macrocycle with a TTF, a 4,4′-bipyridinium and a dihydroxynaphthalene recognition site which is encircled by a cyclobis(paraquat-*p*-phenylene) wheel. In the resting state, the wheel is located at the TTF unit forming a donor–acceptor complex with a green color. Oxidation moves the ring to the second π-electron-rich station, the dihydroxynaphtalene. This donor–acceptor complex has a reddish color. However, in contrast to other bistable catenanes, the third 4,4′-bipyridinium station is also redox-active. Reduction of the system leads to a 4,4′-bipyridinium radical cation which forms a purple trisradical complex with the doubly reduced cyclobis(paraquat-*p*-phenylene). Electrochemical and several spectroscopic techniques showed that, overall, six stable oxidation states – each of them with a unique color – and three co-conformations are accessible in this single compound.

#### 6.2. Switchable catenanes in ordered arrays

**Figure 26:**
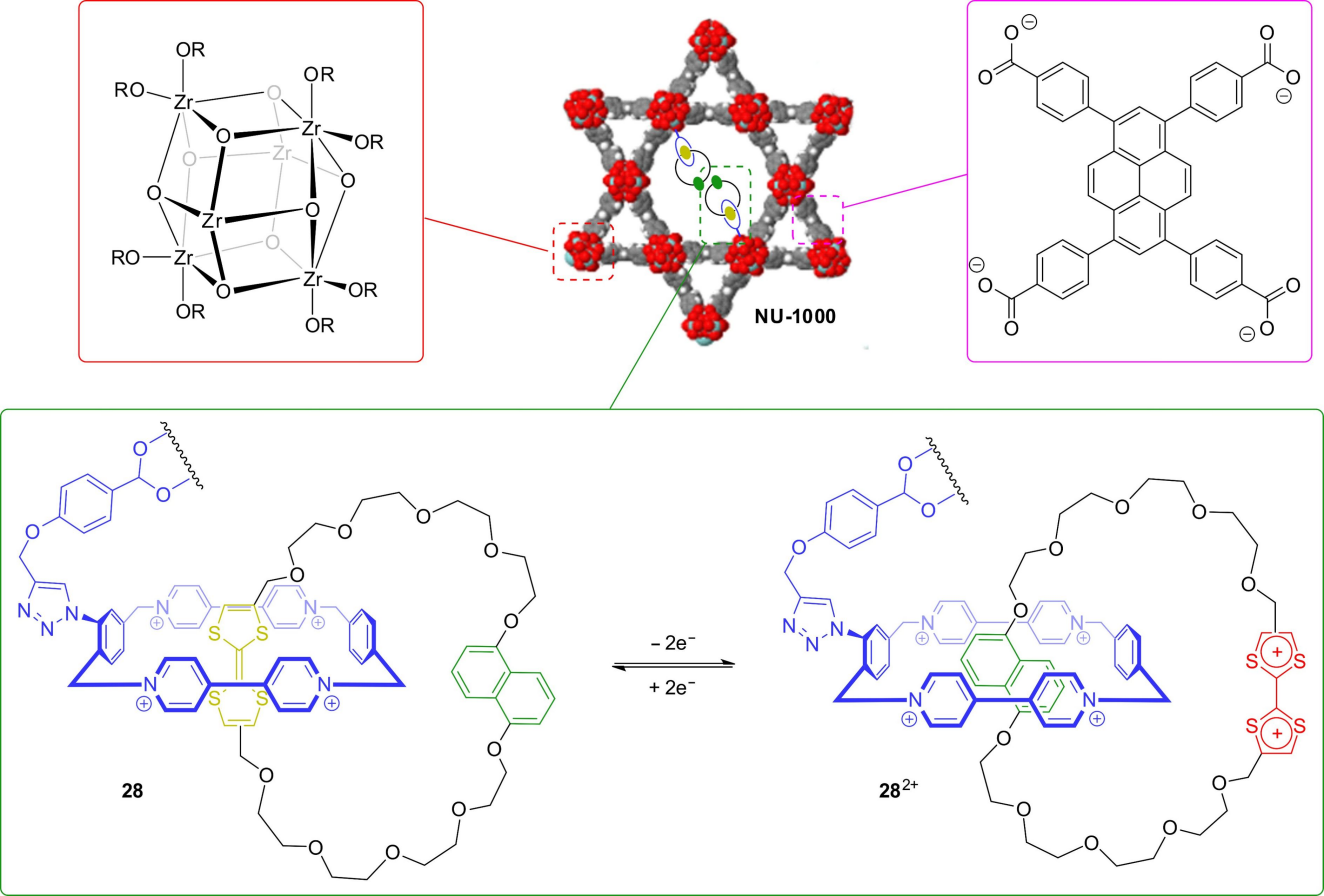
Structure of catenane-functionalized MOF **NU-1000** [108] with structural representation of subcomponents. The TTF-based catenane **28** can be reversibly switched inside the MOF.

Besides ordered arrays on surfaces, on nanoparticles or in Langmuir–Blodgett films, a possibility to arrange bistable catenanes in an ordered fashion is to incorporate them into the rigid scaffold of a metal-organic framework (MOF) [107]. An advantage of this strategy is that the relatively labile organic switches are protected from degradation in this solid material. In 2016, the groups of Hupp, Farha, and Stoddart reported on a bistable donor–acceptor catenane **28** which is inserted in the Zr-based MOF **NU-1000** (Figure 26) [108]. The MOF **NU-1000** consists of Zr_6_ nodes which are bridged by 1,3,6,8-tetrakis(*p*-carboxyphenyl)pyrene ligands [109]. Four hydroxy groups of each metal cluster are pointing into the mesoporous channels of the MOF and can be post-functionalized. Similar to a previous report about rotaxanes implemented into a MOF [110], the catenane was attached to the MOF framework by a so-called solvent-assisted ligand incorporation protocol. A degree of incorporation of ≈0.65 catenanes per Zr_6_ node could be achieved as shown by ^1^H NMR and coupled plasma atomic emission spectroscopy. This degree of functionalization results in a density of ordered catenanes of 8.8 × 10^19^ units cm^−3^ in the MOF. Cyclic voltammetry in combination with chemical oxidation/reduction and powder-UV–vis–NIR spectroscopy showed the catenane to be reversibly switched inside the MOF.

#### 6.3. TTF Dimer interactions in catenanes

**Figure 27:**
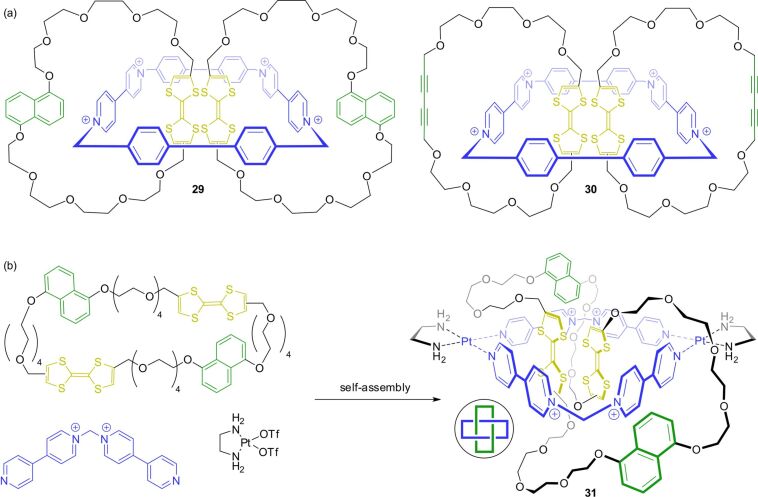
(a) [3]Catenanes **29** and **30** which can stabilize mixed-valence or radical-cation dimers of TTF. (b) Self-assembly synthesis of the molecular Solomon link **31** incorporating two TTF units.

Catenanes are ideally suited structures to enable the formation of TTF dimers, which need a confined molecular space to be stable at room temperature in solution. In 2010, the groups of Cooke and Stoddart described two [3]catenanes consisting of a cyclobis(paraquat-4,4′-biphenylene) and two TTF-based macrocycles (Figure 27a) [111]. Crystal structures of catenanes **29** and **30** showed that both TTF units are in the cavity of the central ring in the unswitched state. During the stepwise oxidation, both catenanes display characteristic spectroscopic features for stable mixed-valence (TTF_2_)^●+^ and radical-cation (TTF^●+^)_2_ dimers. The authors call the stabilizing environment of a [3]catenane a “molecular flask”. However, whereas catenane **29** is directly oxidized from its radical-cation-dimer state (**29**^2(●+)^) to the fully oxidized **29**^4+^ state, the alkyne-based catenane shows a metastable disproportionation equilibrium between the **30**^3+^ state and the **30**^2(●+)^/**30**^4+^ states. The authors explain the discrepancy by the additional binding energy of the dihydroxynaphthalene stations in **29**^4+^ to the cyclobis(paraquat-4,4′-biphenylene) wheel. Therefore, the Coulomb repulsion and the subsequent expulsion of the TTF^2+^ units from the cavity of the central ring lead to a circumrotation of both outer wheels to a co-conformation in which the second binding stations are located in the cavity of the inner wheel.

In a series of similar self-assembled catenanes with a central metallo-supramolecular wheel (catenane **31** is shown exemplarily in Figure 27b), donor–acceptor interactions and hydrogen bonding generate a neutral TTF dimer that is surrounded by cofacially oriented bipyridinium units [112]. The intertwined structure is locked by formation of platinum(II)–pyridine coordination bonds. Interestingly, the doubly interlocked catenane features the topologic structure of a so-called Solomon link. It was shown that the oxidation of the Solomon link to the **31**^●+^ state creates a stabilized mixed-valence dimer (TTF_2_)^●+^. However, in comparison to the structurally similar catenanes in this report, the radical-cation-dimer state (TTF^●+^)_2_ was only transiently stable. The authors suggest an effect of the constrained structural environment of the TTF units, which rationalizes the reduced stability of the radical-cation dimer. Thus, also topological effects have to be considered for TTF dimer formation in redox-switchable MIMs.

## Conclusion

The organosulfur compound TTF developed from a molecular switch with multiple electronic and material applications to one of the most widely used building blocks for the construction of stimuli-responsive MIMs and functional molecular devices. The development of straightforward organic reactions to implement TTFs in rotaxane or catenane structures lead to a variety of different construction motifs. Its high stability in three different oxidation states and the change of multiple properties during these successive oxidations are ideally suited to drive molecular motions in MIMs. Additionally, the optoelectronic and magnetic properties of TTF make it very easy to follow the stimuli-induced motion and the conformational changes accompanying it. TTF dimer interactions are relatively new yet offer an outstanding additional possibility to control molecular motion. In future, the already somewhat explored pathway to ordered arrays of TTF-based AMMs on surfaces or in (Sur)MOFs will enable macroscopic effects caused by concerted electrochemical switching. Furthermore, the disadvantage of degradation of these organic molecules can be potentially overcome by incorporation into more robust materials. The initial dream that AMMs can be used one day to perform different tasks on the molecular level becomes slowly but steadily true. TTF and its derivatives will continue to contribute to this process.
